# Ion-Channel Proteins in the Prepubertal Bitch Reproductive System: The Immunolocalization of ASIC2, ASIC4, and PIEZO2

**DOI:** 10.3390/ijms26094388

**Published:** 2025-05-05

**Authors:** Kamel Mhalhel, Mauro Cavallaro, Lidia Pansera, Gianluca Antonio Franco, Giuseppe Montalbano, Rosaria Laurà, Francesco Abbate, Antonino Germanà, Maria Levanti, Marialuisa Aragona

**Affiliations:** 1 Zebrafish Neuromorphology Lab, Department of Veterinary Sciences, University of Messina, Polo Universitario dell’ Annunziata, 98168 Messina, Italy; kamel.mhalhel@unime.it (K.M.); mauro.cavallaro@unime.it (M.C.); lipansera@unime.it (L.P.); laurar@unime.it (R.L.); abbatef@unime.it (F.A.); antonino.germana@unime.it (A.G.); mblevanti@unime.it (M.L.); 2 Department of Veterinary Sciences, University of Messina, Polo Universitario dell’ Annunziata, 98168 Messina, Italy; gianluca.franco@studenti.unime.it

**Keywords:** ion channels, PIEZO, ASIC, reproductive system, prepubertal stage, bitches

## Abstract

Ion channels play a crucial role in various physiological processes, yet their functions in the reproductive system remain underexplored. This study investigates the expression and the localization of ASIC2, ASIC4, and PIEZO2 ion channels in the reproductive tracts of prepubertal bitches. Western blotting on samples from eight prepubertal bitches confirmed the presence of these ion channels in ovarian, uterine, and uterine tubes tissues, and validated antibody specificity. Immunohistochemistry revealed that all primordial follicles expressed these ion channels, while only some developing follicles showed immunolabeling. These findings suggest ion channels’ potential involvement in oocyte differentiation and maturation. The localization of these channels in uterine tubes, uterine lining, and glandular epithelium suggests a role in tissue maintenance, oocyte transport, and embryo implantation. Additionally, their expression in the tunica media of reproductive vasculature points to a potential role in vascular regulation. Future studies are needed to elucidate the specific mechanisms underlying the role of these channels in reproductive physiology.

## 1. Introduction

The physiology of the bitch reproductive tract is a complex and finely tuned system involving intricate hormonal and physiological interactions that regulate the reproductive cycle and fertility. Research efforts, decorticating those reproductive processes, are necessary to identify novel strategies addressing existing gaps in understanding cellular interactions and their influence on reproductive success.

The prepubertal phase is a critical stage that sets the groundwork for the genital tract’s morphological and functional changes as it prepares for puberty and subsequent reproductive activity. The reproductive system begins to mature during this period, exhibiting initial hormonal shifts and structural adaptations [1,2].

Understanding bitches’ reproductive developmental changes during this window is essential for optimizing breeding management, ensuring reproductive health, and providing comparative data for other species. However, few comprehensive studies have investigated the physiological transformations that bitches undergo during this stage [3]. Various factors contribute to mammalian reproductive physiology, including growth factors [4], hormones, and ion channels [5,6].

Ion channels are essential membrane proteins that allow the selective stream of ions across cellular membranes, critically influencing a range of physiological processes. Their gating can be triggered by various mechanisms, including micro-environmental pH changes, namely in the case of ASICs (acid-sensing ion channels), or mechanical-induced membrane potential changes for PIEZO (from the Greek “piezein” meaning to press or squeeze; mechanosensitive ion channels) proteins including PIEZO2, relevant to reproductive processes. Sex steroid hormones (SSHs) including estrogens and androgens may modulate the expression of various ion channels from hormone-sensitive tissues, including the breast, ovary, and cervix. SSH can regulate ion-channel activity through the canonical transcriptional pathway [7,8], or bind to channel domains and in turn induce the expression of ion-channel-interacting proteins, thereby altering channel-gating properties, resulting in rapid cellular responses [9,10]. For example, the mRNA levels of potassium voltage-gated channel subfamily H member 1 (Kv10.1) were up-regulated by estradiol in primary cultures from human vascular endothelial cells (HUVEC cell line), and this effect was abolished by an estrogen receptor alpha antagonist. Moreover, potassium currents resembling Kv10.1 channel activity were amplified in the cervix by estradiol treatment [11]. Another example of ion channels’ regulation by SSH is the estrogen-mediated modulation of Ca^2+^ intake through the TRPV6 calcium ion channels, assessed on human T84 colonic cells [12]. Additionally, the molecular mechanisms underlying the inhibition of chloride secretion by estrogen have been described. This process involves the modulation of the potassium voltage-gated channel subfamily Q member 1 (KCNQ1): and its regulatory subunit (KCNE3) through Protein Kinase A catalytic subunit isoform I PKACI activation, and subsequent KCNE3 phosphorylation [13,14].

Despite the importance of the prepubertal developmental stage, the few available studies assessing the expression of ion channels in the genital tract have been conducted on adult animals. Indeed, a study on several transient receptor potential (TRP) channels have been undertaken in the uterus of a sexually mature bovine. This study revealed the cyclic expression of TRPC 1, 2, 4, and 6 isoforms in the uterine [15] and oviductal epithelium throughout the estrous cycle [16]. In mice, the expression of TRPV6 was proved in uterus and its expression seems to increase at estrus and fluctuate during pregnancy [8]. Additionally, TRP vanilloid 4 (TRPV4) was detected in motile cilia on the epithelial cells of the murine ampulla and isthmus [17].

Although many of these channels are located at the plasma membrane, intracellular and intercellular channels ensure ion-channel influx, crucial for cellular processes during oocyte maturation and egg activation.

ASICs, members of the DEG/ENaC (DEGenerin/Epithelial Na^+^ Channel) superfamily, are Na^+^-permeable ion channels activated by extracellular H^+^ in response to a rapid drop in environmental pH [18,19]. Structurally, ASICs consist of two transmembrane domains (TMD1 and TMD2), a large extracellular loop containing 14 conserved cysteines, and short cytoplasmic N- and C-terminal regions [20]. In mammals, six ASIC proteins, defined as ASIC1a, ASIC1b, ASIC2a, ASIC2b, ASIC3, and ASIC4, are encoded by four genes [21]. A seventh subunit, ASIC5, known as NaC, or BASIC, is encoded by Asic5. It is the most divergent member of the Asics family. The Asic5 gene was identified in many species, including dog (Gene ID: 482664) [22]. The mechanically activated PIEZO channels identified in 2010, however, represent a class of two large proteins, PIEZO1 and PIEZO2, selectively allowing for the influx of cations [23]. Each PIEZO protein comprises over 2000 amino acids, forming trimeric complexes with a propeller-like structure consisting of three blades arranged around a central pore [24]. This unique architecture allows PIEZO channels to sense and respond to different levels of membrane tension, maintaining functionality under mechanical stress [25,26]. When activated, these channels produce non-selective cationic currents that conduct the inward flow of cationic ions, especially Ca^2+^, generating electrical signals transmitted to the nervous system [27]. Members of both ion channels’ families are key mediators of several physiological processes including chemical [28] and mechanical [29] perception, muscle contraction [30,31], and arterial baroreception reflex [32,33]. They have been encountered in different types of epithelia [34,35], including those found in the oviduct and epididymis [36]. Moreover, previous studies in other species, including the *Caenorhabditis elegans*, *Dicentrarchus labrax*, and Sprague Dawley rats’ strain, have demonstrated the potential involvement of these ion channels in gonadal differentiation and gamete development, as well as their ability to coordinate activity across multiple reproductive tissues. These findings support the hypothesis that ASICs, including ASIC2 and ASIC4 and PIEZO channels, including PIEZO2, may play significant roles in reproductive function. Additionally, there are limited bibliographical data regarding the role of ion channels in reproductive physiology across animal species and a notable gap in research specifically concerning the canine reproductive tract. While some studies have investigated other ion channels in bovine and murine models, to our knowledge, no studies have assessed ASIC2, ASIC4, or PIEZO2 in the genital tract of bitches.

In light of the above-mentioned literature and the scarcity of bibliographical data on the ion channels’ potential role in canine genital tracts, the current study aimed to investigate, for the first time, the presence of ASIC2, ASIC4, and PIEZO2 ion channels in the ovary, oviducts, and uterus of prepubertal bitches, decorticating its possible implications on the reproductive apparatus during this critical developmental period, and establish the distribution of these ion channels in canine reproductive tissues without the influence of hormonal fluctuations associated with puberty and reproductive cycles.

## 2. Results

### 2.1. Ion Channels’ Evolutionary Conservation

The evolutionary perspective is increasingly being adopted as essential for emphasizing the key role of the conserved proteins in the physiological process among different species. Thus, in the current study, the comparative/evolutionary perspective has been adopted in order to evaluate the conserveness of the here-studied ion channels to draw some general conclusions with respect to more and less divergent species. Indeed, the acid-sensing ion channels ASIC2 and ASIC4 as well as PIEZO-type mechanosensitive ion-channel component 2 PIEZO2 from dogs, humans, mice, green anole, chicken, and zebrafish have been checked for sequence similarities. The ASIC2 from mice was 98.83% similar to its canine ortholog compared to 87.18%, 78.15%, 76.80%, and 71.67% in green anole, humans, chicken and zebrafish, respectively. Same for ASIC4, for which *Mus musculus*, and *Homo sapiens* had the highest homology to the canine protein (93.51% and 97.40%, respectively) while *Anolis carolinensis*, *Gallus gallus*, and *Danio rerio* have a more divergent protein to canine ASIC4; still, the homology was determined to be between 62.82% and 73.11%. Finally, the human and mice PIEZO2 were more than 90% similar to its canine ortholog, while those of chicken, green anole, and zebrafish were 79.72%, 74.84%, and 62.89% similar (Table 1, Supplementary Material: Figures S1–S3).

The multiple ASIC2 protein alignment (Supplementary Material: Figures S1–S3) highlights the conservation of the first extracellular topological domain, the second cytoplasmic topological domain, and the second helical transmembrane domain among the five key representative taxa from major vertebrate groups.

### 2.2. Histological Organization of the Canine Prepubertal Genital Tracts

**Figure 1:**
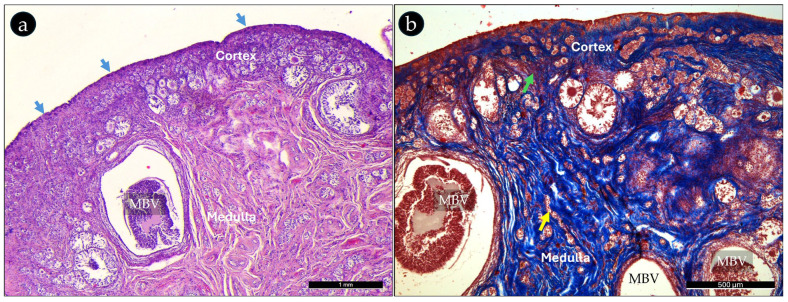
Photomicrographs of (**a**) H&E- and (**b**) Masson’s trichrome-stained cross-section of the prepubertal bitch’s ovary (low magnification). The overall organization of the ovary in cortex and medulla can be observed beside the arrangement of the capsule of ovary (blue arrows), and subsurface epithelial structures. Interstitial cells (green arrow); ovarian medullary blood vessels (MBV); rete ovarii (yellow arow). Magnification 4× (**a**); 10× (**b**).

The histological analysis of bitches’ reproductive systems provides a microscopic examination of the cellular structure and organization of key reproductive organs, including the ovaries, the uterine tubes, and uterus, highlighting their distinct tissue (Figure 1).

The ovary was made of cortex and medulla. The cortical parenchyma was composed of follicles, interstitial cells, and collagenous connective tissue stroma (Figure 1b), covered by cuboidal epithelium (Figure 1). The ovarian medulla contains large blood vessels (ovarian artery branches, and vein tributaries), and lymphatics embedded in a loose collagenous matrix. An epithelial network of irregular tubules and cords, the rete ovarii, was also identified.

**Figure 2:**
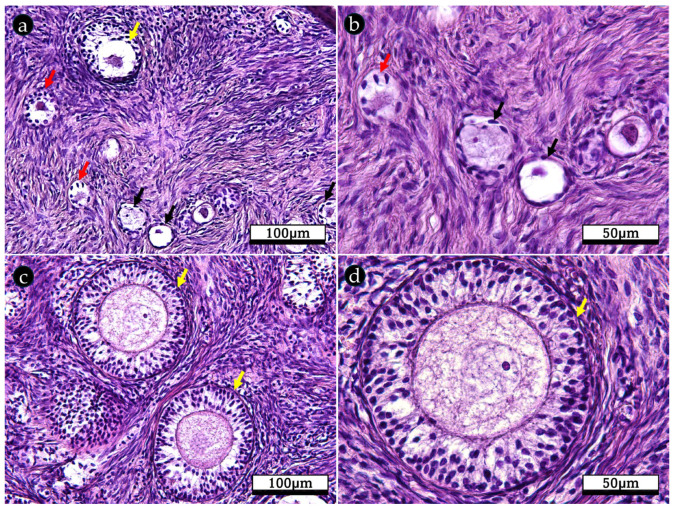
Photomicrographs of a H&E-stained ovary of a prepubertal bitch. (**a**,**b**) Normal primordial (black arrows), primary (red arrow), and early secondary (yellow arrow) follicles. (**c**,**d**) Secondary follicles (yellow arrow), with central oocyte and the surrounding layer of granulosa cells, within the ovarian stroma. Magnification 20× (**a**,**c**); 40× (**b**,**d**).

The ovarian cortex follicle development is a sequential process. Thus, follicles developed from primordial follicles to primary and secondary follicles. Primordial follicles contained a primary oocyte surrounded by one layer of flattened follicular cells (Figure 2a,b). Primary follicles, containing an oocyte surrounded by cuboidal follicular cells known as granulosa cells (Figure 2a,b), were bordered by an organized stratum of stromal cells called theca cells. Secondary follicles, however, were characterized by multiple layers of granulosa cells and two layers of stromal cells, the theca interna and externa (Figure 2c,d).

Each ovary was interfaced with the uterine tubes, which transport the oocyte towards the uterine horns. The histological organization of fimbriae, isthmus, and uterus as representative tracts of the uterine tubes was described below.

**Figure 3:**
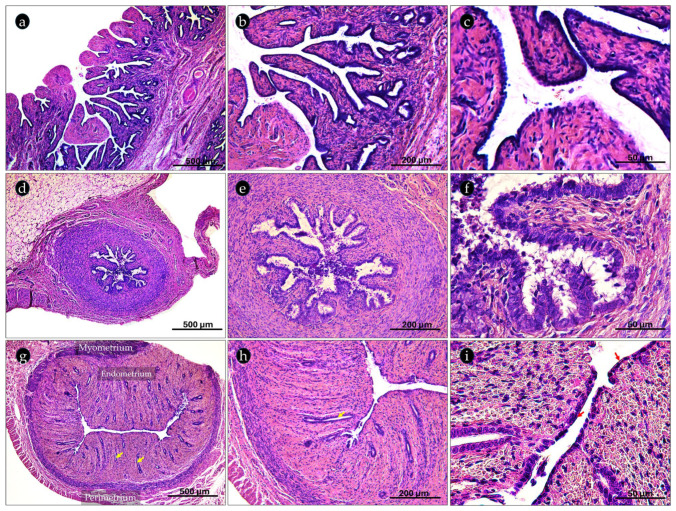
Photomicrographs of H&E-stained sections of prepubertal bitches’ uterine tubes. (**a**–**c**) depict the fimbriae, (**d**–**f**) show the isthmus, and (**g**–**i**) represent the uterus. The uterine cuboidal epithelium is indicated by the red arrow, while the uterine glands are marked by the yellow arrow. Magnification 10× (**a**,**d**,**g**); 20× (**b**,**e**,**h**); 40× (**c**,**f**,**i**).

The fimbriae coronated the distal end of the infundibulum. It contains many developed mucosal folds, covered by a columnar epithelium, that will guide the ovulated oocyte into the uterine tube (Figure 3a–c). As the oviduct progresses toward the isthmus, the mucosal folds become less intricate and the myosalpinx, the muscular layer, however, becomes thicker and well developed (Figure 3d). The isthmus was lined with a pseudostratified columnar epithelium with both ciliated and non-ciliated cells (Figure 3e,f), while the cross-sectional anatomy of the uterus is divided into the endometrium, the myometrium, and the perimetrium (Figure 3g,h). The endometrium was covered with simple cuboidal epithelium (Figure 3i), which rests on a loose lamina propria. Beneath the mucosa, several tubular shallow uterine glands expand from the lamina propria to the submucosa (Figure 3g–i). The myometrium, however, was composed of smooth circular muscle layer (Figure 3g,h). The perimetrium, the uterine outermost layer, was composed of loose connective tissue, and small arteries and veins with an overlying mesothelial surface.

### 2.3. Ion-Channel Expression and Primary Antibody Specificity in Canine Sample

The anti-ASIC2, ASIC4, and PIEZO2 antibodies are raised against peptides synthesized from their respective human proteins. The alignment of the anti-ASIC 2, ASIC 4, and PIEZO2 immunogen sequences from human proteins and their respective sequences from bitches revealed an identity of 100%, 98.33%, and 92%, respectively (Table 2). The high identity between the antibody immunogens and the respective bitches’ sequences allowed us to hypothesize that the used commercial antibody could be effective on bitches.

**Figure 4:**
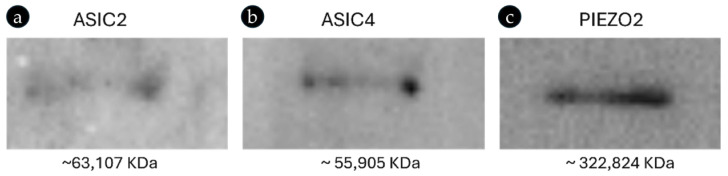
Western blot analyses showing bands corresponding to the molecular weights of the bitches’ ASIC2 (**a**), ASIC4 (**b**), and PIEZO2 (**c**) proteins.

The expression of ASIC2, ASIC4, and PIEZO2 ion channels in bitches’ genital tracts (ovary, uterus, and uterine tubes), as well as the specificity of the antibodies used here, were further validated using Western blot analyses. The blots of the bitches’ proteins incubated with the above-mentioned antibodies (see Table 2) revealed bands of approximately 63,107, 55,905, and 322,824 KDa (Figure 4), corresponding to the molecular weights of the bitches’ ASIC2, ASIC4, and PIEZO2 proteins, respectively, confirming the presence of ASIC2, ASIC4, and PIEZO2 proteins in the ovarian, uterine, and uterine tube tissues of bitches and the specificity of the used antibodies to canine proteins.

### 2.4. ASIC2, ASIC4 and PIEZO2 Localization in the Bitches’ Genital Tracts

**Figure 5:**
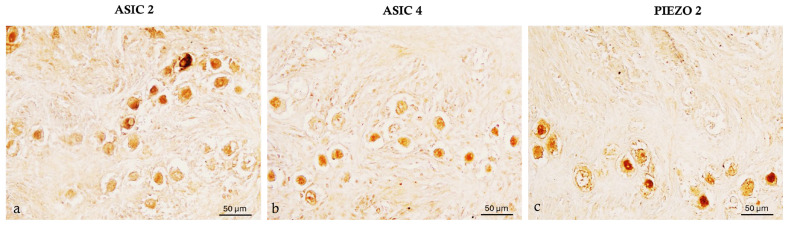
Photomicrographs of ASIC2, ASIC4, and PIEZO2 immunoreactivity in the ovary of prepubertal bitches. (**a**) ASIC 2 immunoreactive primordial follicles; (**b**) ASIC4 immunopositive primordial follicles; (**c**) PIEZO2 immunolabeled primordial follicles. Magnification 40×.

**Figure 6:**
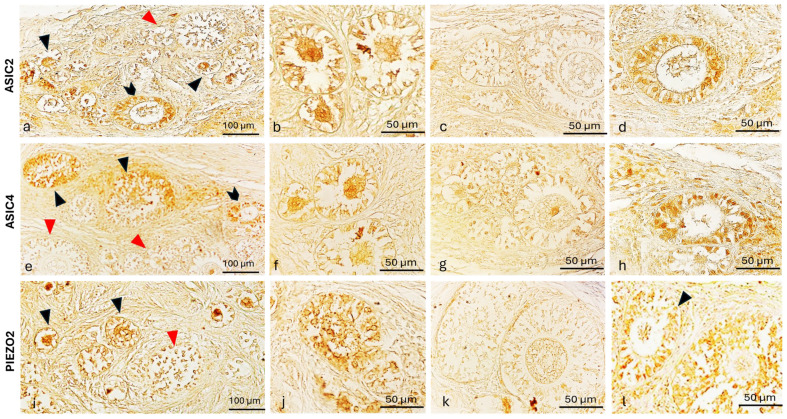
Photomicrographs of ASIC2, ASIC4, and PIEZO2 immunoreactivity in the ovary of a prepubertal bitch. (**a**) ASIC2 immunolabelled oocyte of primary (arrowheads) and granulosa cells of secondary (chevron arrow) follicles beside the ASIC2 non-reactive primary follicles (red arrowhead); (**b**) ASIC2 immunopositive oocytes and granulosa cells of primary follicles; (**c**) ASIC2 non-immunolabelled primary follicles; (**d**) ASIC2 immunoreactive granulosa cells of secondary follicles; (**e**) ASIC4 immunoreactive granulosa cells of primary (arrowheads) and secondary (chevron arrow) follicles beside the ASIC4 non-immunolabelled primary follicles (red arrowheads), (**f**) ASIC4 immunopositive oocyte and granulosa cells of primary follicles; (**g**) ASIC4 non-immunoreactive secondary follicles; (**h**) ASIC4 immunolabeled granulosa of secondary follicle; (**i**) PIEZO2 immunoreactive oocyte and granulosa cells (arrowheads) in primary follicles and non-immunoreactive (red arrowhead) primary follicle; (**j**) PIEZO2 immunolabelled granulosa cells of primary follicles; (**k**) PIEZO2 non-immunoreactive primary follicles; (**l**) PIEZO2 immunolabelled granulosa cells of secondary follicles (arrowhead). Magnification 20× (**a**,**e**,**i**); 40× (**b**–**d**,**f**–**h**,**j**–**l**).

An immunohistochemical analysis was carried out on serial sections of the ovary, uterus, and uterine tubes of prepubertal bitches using the peroxidase method. Positive cells were identified, based on a morpho-topographical approach, revealing ASIC2, ASIC4, and PIEZO2 immunoreactivity in the studied segments of the prepubertal bitch reproductive system. In the ovary, the follicles reacted differentially to the here-used antibody during development (Figure 5 and Figure 6). Indeed, the primordial follicles were ASIC2 (Figure 5a), ASIC4 (Figure 5b), and PIEZO2 (Figure 5c) immunolabeled.

**Figure 7:**
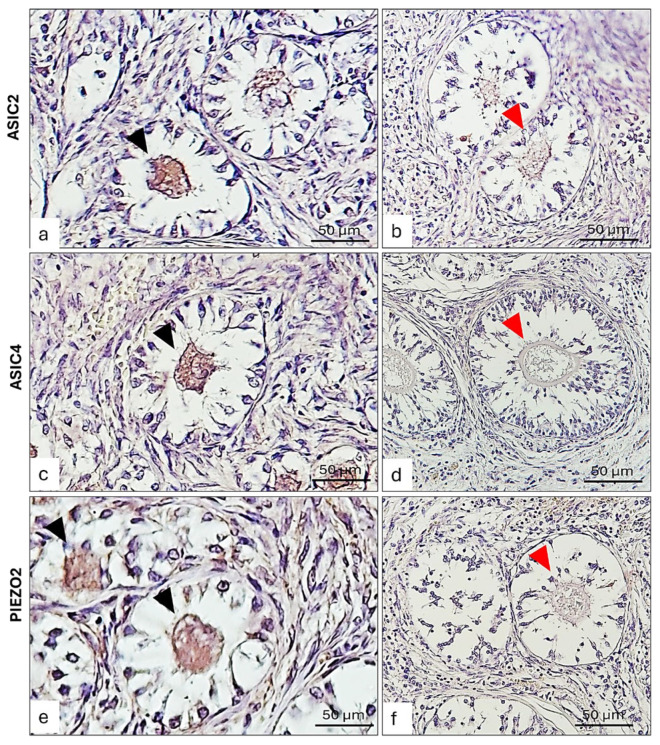
Photomicrographs of ASIC2, ASIC4, and PIEZO2 immunolabelling, counterstained with hematoxylin, in bitches’ ovaries. (**a**) ASIC2 immunolabelled oocyte of primary follicles (arrowheads); (**b**) ASIC2 non-reactive oocyte in primary follicles (red arrowhead); (**c**) ASIC4 immunopositive oocytes (arrowhead) of primary follicles; (**d**) ASIC4 non-immunolabelled oocyte (red arrowhead) in primary follicles; (**e**) PIEZO2 immunoreactive oocyte (arrowhead) of primary follicles; (**f**) PIEZO2 non-immunoreactive oocyte (red arrowhead) in primary follicles. Magnification 40×.

**Figure 8:**
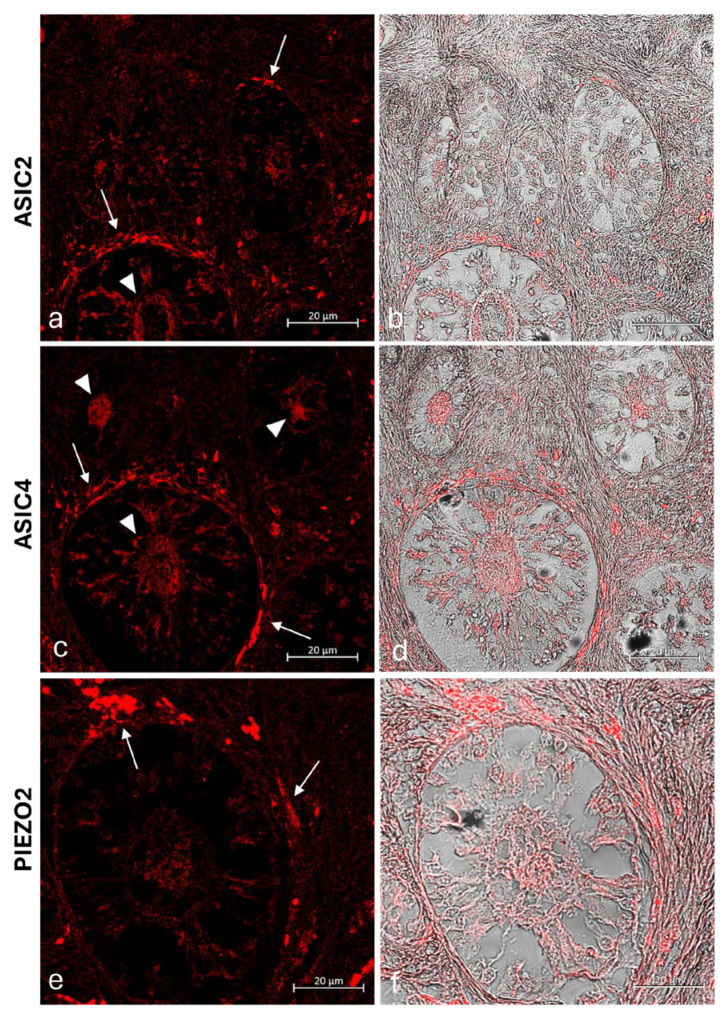
Transmitted light and immunofluorescence photomicrographs of ASIC2, ASIC4, and PIEZO2 immunoreactivity in the ovary of a prepubertal bitch. (**a**) ASIC2 immunolabelled oocyte (arrowheads) and theca cells (arrows) of primary follicles; (**b**) corresponding transmitted light and immunofluorescence merged confocal micrographs of section (**a**); (**c**) ASIC4 immunolabelled oocyte (arrowheads) and theca cells (arrows) of primary follicles; (**d**) corresponding transmitted light and immunofluorescence merged confocal micrographs of section (**c**); (**e**) PIEZO2 immunoreactive theca cells (arrows) of primary follicles; (**f**) corresponding transmitted light and immunofluorescence merged confocal micrograph of section (**e**); magnification 40×.

However, the growing follicles showed different spatial-distribution mapping for the examined antibodies. Some primary follicles have the oocyte and the surrounding granulosa cells ASIC2, ASIC4, and PIEZO2 immunolabeled (Figure 6a,b,e,f,i,j), while others were not immunoreactive in any part (Figure 6a,c,e,j,i,k). To better contrast the primary follicles’ structures immunoreactivity, further immunohistochemistry reactions counterstained with hematoxylin (Figure 7), and immunofluorescences reactions taken from specific planes with confocal microscope (Figure 8) have been conducted, reporting a labeled theca (Figure 8). Though, all the secondary follicles were ASIC2, ASIC4, and PIEZO2 immunopositive (Figure 6d,h,l).

**Figure 9:**
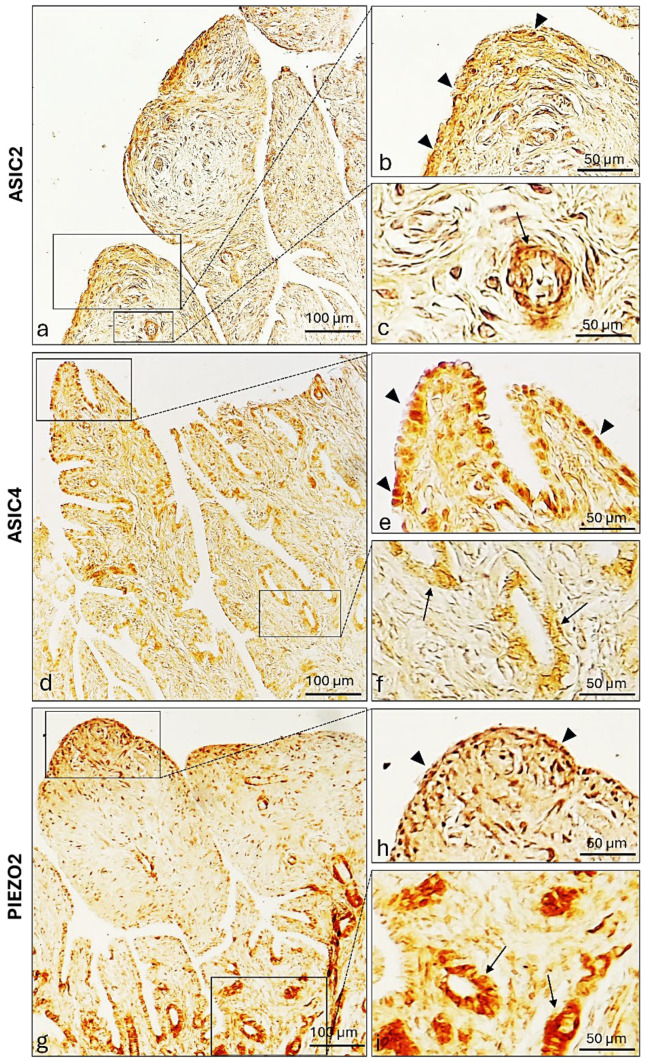
Photomicrographs of ASIC2, ASIC4, and PIEZO2 immunoreactivity in the fimbriae of a prepubertal bitch (**a**–**c**). (**b**) ASIC2, (**e**) ASIC4, and (**h**) PIEZO2 immunolabeled fimbrial lining epithelium (arrowheads). (**c**) ASIC2, (**f**) ASIC4, and (**i**) PIEZO2 immunoreactive fimbrial glandular epithelium (arrows). Magnification 20× (**a**,**d**,**g**); 40× (**b**,**c**,**e**,**f**,**h**,**i**).

ASIC2, ASIC4, and PIEZO2 immunopositivity were also investigated in other parts of the prepubertal bitches’ genital apparatus. Particularly, immunoreactivity was observed—in both fimbrial lining and glandular epithelium (Figure 9).

**Figure 10:**
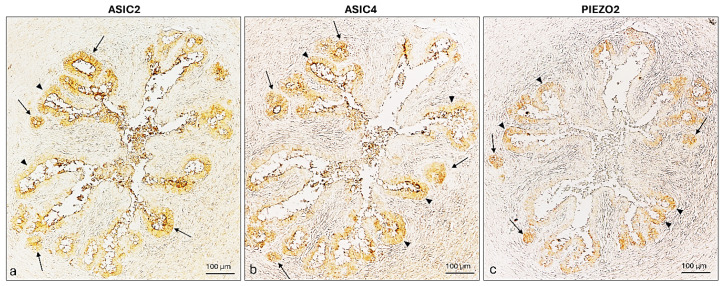
ASIC2, ASIC4, and PIEZO2 immunoreactivity in the isthmus of a prepubertal bitch. (**a**) ASIC2, (**b**) ASIC4, (**c**) PIEZO2 immunolocalization in the lining (arrowheads) and glandular (arrows) epithelium. Magnification 20×.

An immunohistochemical analysis of ASIC2, ASIC4, and PIEZO2, performed independently on separate tissue sections, demonstrated the presence of all three ion-channel proteins in the isthmus where both lining and glandular epitheliums were immunopositive (Figure 10).

**Figure 11:**
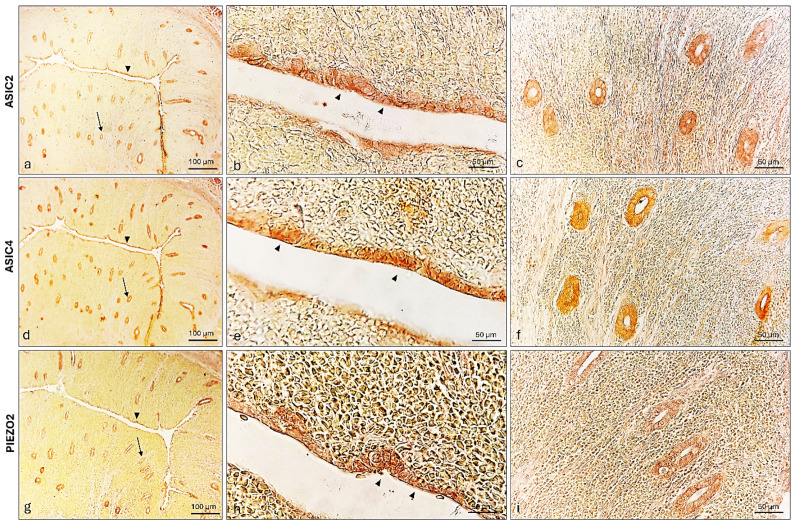
ASIC2 (**a**), ASIC4 (**d**), and PIEZO2 (**g**) immunoreactivity in the uterine lining, epithelium (arrowhead), and glands (arrow) of a prepubertal bitch. (**b**) ASIC2 immunopositive lining of the epithelium (arrowheads); (**c**) high magnification of ASIC2 immunoreactive uterine glands; (**e**) ASIC4 immunoreactive lining of the epithelium (arrowheads); (**f**) ASIC4 immunoreactivity in uterine glands, (**h**) PIEZO2 immunopositive lining of the epithelium (arrowheads); (**i**) PIEZO2 immunolabelled uterine glands. Magnification 20× (**a**,**d**,**g**); 40× (**b**,**c**,**e**,**f**,**h**,**i**).

In the prepuberal bitch uterus, the lining endometrium was ASIC2, ASIC4, and PIEZO2 immunoreactive (Figure 11a,b,d,e,g,h). Moreover, the three ion channels have also been detected in the glandular epithelium (Figure 11c,f,i).

**Figure 12:**
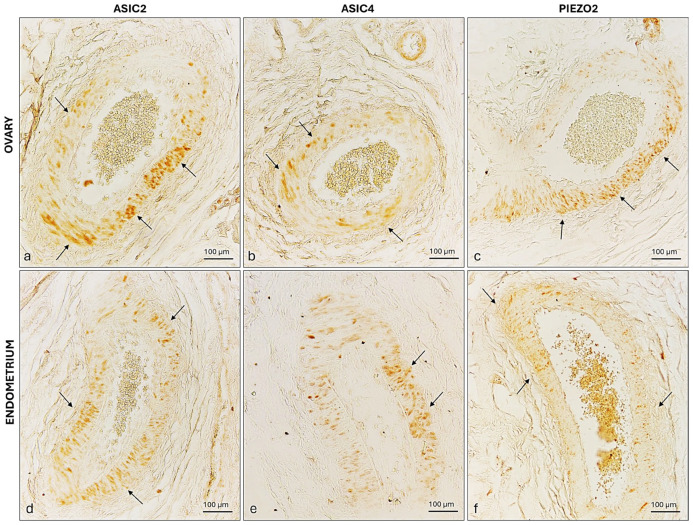
ASIC2, ASIC 4, and PIEZO2 immunoreactivity in the ovarian and endometrium blood vessels of a prepubertal bitch. (**a**) ASIC2, (**b**) ASIC4, and (**c**) PIEZO2 immunoreactivity in the tunica media (arrows) of the prepubertal bitch’s ovarian blood vessel. (**d**) ASIC2, (**e**) ASIC4, and (**f**) PIEZO2 immunoreactivity in the tunica media (arrows) of the prepubertal bitch’s endometrium blood vessel. Magnification 20×.

Finally, the tunica media of the prepubertal bitch ovary and endometrium blood vessels were immunoreactive to the three ion channels (Figure 12).

## 3. Discussion

Studying canine reproductive physiology is crucial for veterinary medicine, breeding practices, and scientific innovation. Indeed, an in-depth understanding of the bitches’ reproductive system is crucial to ensure healthy and successful breeding, support the diagnosis and treatment of reproductive disorders, and enhance fertility. Moreover, research in this field could drive advancements in reproductive technologies, leading to improved outcomes in natural and assisted breeding methods.

Bitch development stages can be divided into the neonatal and transitional stage (~12–14), the socialization period (3 weeks), the prepubertal period (4 months to 6 months), and the sexual maturity stage (6 months to 12 months) [2]. The prepubertal period represents a critical stage that sets the ground for the genital tract’s morphological and functional changes as it prepares for puberty and subsequent reproductive activity. Only a few fundamental studies in the literature have evaluated the process of physiological changes by which domestic canids mature into adults capable of sexual reproduction [3,37,38,39].

A considerable number of intricately interlinked factors are involved in mammalian reproductive physiology, among which are the plasma membrane and intra and intercellular ion channels [5,6,40,41]. Understanding ion-channel expression during the prepubertal period could provide valuable insights into the developmental physiology of the reproductive system, identifying key regulatory mechanisms that govern the transition to sexual maturity.

A comparative genome analysis shows that most human ion-channel gene families originated in the earliest metazoans [42]. In the current study, the high homology of humans, mice, and even the most divergent vertebrate groups’ ASIC2 and ASIC4 compared to dogs’ ortholog highlights the strong evolutionary conservation of these ion channels despite the adaptative divergence among the groups [43]. Similarly to ASICs, PIEZO2 exhibits remarkable conservation among mammals. This level of conservation suggests that PIEZO2 ensures a critical function preserved in mammalian lineages, as postulated by Thomas Meinel: Similar proteins possess with high probability a common ancestry and therefore a similar function [44]. The multiple-sequence alignment reveals the conservation of ASIC2’s extracellular and cytoplasmic topological domains, and the second helical transmembrane domains known to play essential roles in ion-channel gating and signal transduction. This study’s comparative and evolutionary approach demonstrates ion-channel preservation among vertebrates, underscoring their fundamental roles.

The authors have started by studying the histological organization of the ovary and different tracts of the oviducts going through the fimbriae, isthmus, and uterus. The here-described histological architecture of the prepubertal bitches’ ovary was comparable to those of mature bitches described previously in the bibliography, except for the characteristics of the prepubertal stage, namely the lack of the Graafian follicles or the shallow uterine tubular gland [37,45,46,47,48]. Moreover, the mature bitches, however, have a thick and more glandular uterine body, with a different structure of the endometrium depending on the current stage of the estrous cycle [37].

Comparative histological analysis suggests that many features of the canine ovary and uterus are conserved among mammals. The stages of canine folliculogenesis closely mirror those observed in rodents and humans [49,50]. Similarly, the structure of the endometrial lining and uterine glands share functional roles in supporting fertilization and implantation, regulated by hormonal status [51]. These histological parallels indicate conserved reproductive strategies among mammals.

Moreover, authors have assessed for the first time the expression and localization of ASIC2, ASIC4, and PIEZO2 ion channels in the genital-tract tissues of prepubertal bitches. The findings were discussed in the context of the limited available literature on other species, and established the distribution of these ion channels in canine reproductive tissues without the influence of hormonal fluctuations associated with puberty and reproductive cycles., providing hypothetical insights into the potential roles of these channels in the critical prepubertal stage.

We first verified high-sequence homology between the immunogen peptides and the corresponding canine sequences to confirm antibody specificity for canine tissues. This similarity supports cross-reactivity. Additionally, Western blot analysis revealed bands at molecular weights consistent with ASIC2, ASIC4, and PIEZO2, further validating antibody specificity. These findings support the consistency of the subsequent immunohistochemistry results. The immunohistochemistry investigation revealed for the first time the expression of the three ion channels in the ovarian epithelium, in all primordial follicles, as well as in some primary and secondary follicles. These findings bear similarity to those reported in other species, in that those ion channels were expressed, to the authors’ best knowledge, in follicular structures of phylogenetically distant species. Indeed, the expression of both ASIC2 and ASIC4 has been proved in European seabass previtellogenic oocytes and in scattered intratesticular previtellogenic oocyte cells within testis [52]. The detection of the here-studied ion channels in all the primordial follicles could be explained by their implication in maintaining the germinal line stock. In the ovarian environment, shifts in pH sensed by ASICs could regulate cellular responses. In fact, ASICs’ acidic activation in ovarian tissue could induce a remodeling of the cellular homeostasis as proved in colorectal cancer-cell studies, where the up-regulation of the ASIC2 significantly enhanced cell proliferation via the calcineurin/NFAT1 signaling pathway, while ASIC2 knockdown had the reverse effect [53,54]. The differential expression of ion channels within follicles at the same stage, however, can be attributed to several factors, including physiological differences arising from follicular recruitment and selection processes. During follicular recruitment, only certain follicles are primed to continue development, while others may not express the same markers due to varying levels of hormonal influence and cellular interactions, and may undergo atresia. The expression of ASICs and PIEZO ion channels reported in the current study in three types of follicles may be explained by their implications in the differentiation and maturation of germ cells. Specifically, during the meiotic arrest at prophase I, oocytes grow and accumulate macromolecular components. Upon stimulation, they undergo a complex differentiation pathway that prepares the eggs for fertilization and the egg-to-embryo transition, where the plasma membrane ion channels’ ionic conductance plays essential roles [55].

Additionally, regulating the ionic environment, ASIC2, and ASIC4 may affect the intracellular calcium concentration [54,56], thereby influencing the calcium-dependent maturation-promoting factor (MPF) pathway and differentially controlling the maturation of the egg, in turn [57,58]. MPF, the master regulator of both the meiotic and mitotic cell cycle, is composed of cyclin-dependent kinase 1 (Cdk1), in complex with cyclin B, and the associated nuclear kinase microtubule-associated threonine-like kinase [41]. Oocyte maturation is complete when the oocyte reaches the arrest in the metaphase of meiosis II, which requires inhibiting the anaphase-promoting complex (APC) and the anaphase II progression prevention by the cytostatic factor. The APC tags regulatory proteins inactivating Cdk1 and thus allowing progression to anaphase [41,58]. Therefore, oocyte maturation is a cellular differentiation program where ionic conductance plays crucial roles.

Comparative studies across different species provide valuable insights into the mechanisms regulating ion channels in gametes, emphasizing the critical role of calcium in oocyte function and suggesting the potential conservation of the underlying pathways involved [59,60,61,62,63].

Compared to ASICs, PIEZO2, as a non-selective cation channel, has higher calcium permeability, allowing for a significant amount of calcium influx [64,65,66]. Induced by several mechanical stimuli within the ovary such as follicular growth and rupture, PIEZO2 could efficiently trigger the MPF and calcineurin/NFAT1 signaling pathways.

Other than the ovary and its different follicles, the here-used subset of antibodies immunolabeling the different segments of the genital tract, including uterine tubes, uterus lining, and glandular epitheliums, brings up the ion channels’ potential implication in epithelial cells’ turnover. Indeed, it was proved that the fast epithelial cells’ sustaining could be activated by the stretch-activated channel PIEZO, which in turn regulates ERK1/2-mediated cell-cycle progression from the G2 to the mitotic (M) phase, initiating mitosis, Ca^2+^-flux-mediated cell proliferation, or cell extrusion [67,68,69]. Additionally, uterine epithelium ion channel labeling has highlighted ion channels’ potential involvement in embryo implantation processes, which requires an intimate interaction between a competent blastocyst and the endometrial epithelial cells. Indeed, ultrastructural animal studies of implantation have demonstrated a physical interaction between the embryo and the endometrial epithelium [70], inducing decidualization, the progesterone-dependent differentiation of fibroblast-like endometrial stromal cells into large, secreting decidual cells. The author’s hypothesis was supported by a study conducted in both humans and mice endometrial epithelial cells (EECs), where the mechanical stimulation, by poking of the cell membrane, induced an increase in current density and amplitude as well as a Ca^2+^ influx proportional to the poking depth. However, the EEC mechanosensitivity was abolished in the presence of GsMTx4, a peptide widely used to block mechanically activated channels [71]. Moreover, the application of the selective chemical agonist of the PIEZO channel, Yoda130, induced a reversible robust increase in intracellular Ca^2+^, outwardly rectifying current densities [72].

Additionally, ASIC2, ASIC4, and PIEZO2 expression on the genital tract’s epithelium may reflect their potential contribution in oocyte migration. Several factors typically influence this process, including ciliary movement within the uterine tube and muscle contractions in the oviduct [73,74,75]. PIEZO family members have already been identified in human endometrium [70], and their role in relaxing [20,76] or contracting [77] smooth muscle depending on their tissue location and the presence of pathology was proved [78]. In fact, previous studies have been conducted in the role of the membrane potential and ion channels on phasic smooth-muscle intermittent contractile activity, including those of the genital tracts [73,79].

In smooth-muscle contraction, excitation–contraction coupling starts with the generation of an action potential increasing the free Ca^2+^ in the cytoplasm, which was modulated by the coordinated action of sodium (Na^+^), chloride (Cl^−^), and potassium (K^+^) ion channels, neurotransmitters, and hormonal signals, leading to cyclic changes in genital-tract tone and elasticity [80,81,82].

Moreover, different studies have reported the distribution of ion channels, namely ENaC, TRPV4, PKD1, and PKD2, on epithelial cells with motile cilia lining the female reproductive tract, all along the oviduct and in the uterine glands where their potential implication on successful oocyte transport has been suggested already [17,74,75,83,84].

Another crucial point to consider is the expression of PIEZO and ASIC ion channels encountered in the tunica media of the bitches’ ovarian and endometrium blood vessels. This finding could be in agreement with the study of Arishe et al., conducted on Sprague Dawley rats, where PIEZO1 activation, using the endothelial nitric oxide-mediated Yoda 1, induced vasorelaxation [22]. ASIC2, however, has been proven to be an important determinant of the arterial baroreceptor reflex. They are expressed in aortic baroreceptor neurons in the nodose ganglia and their terminals [85,86,87].

## 4. Materials and Methods

### 4.1. Evolutionary Conservation of Ion Channels Across Vertebrates: From High Sequence Homology to Potential Critical Function

Protein sequences for ASIC2, ASIC4, and PIEZO2 from five vertebrate taxa—*Homo sapiens* (human), *Mus musculus* (mouse), *Canis lupus familiaris* (dog), *Gallus gallus* (chicken), and *Danio rerio* (zebrafish)—were obtained from previously published entries in the GenBank database (Table 1). All protein sequences were aligned using the default parameters of NCBI BLASTp (protein–protein BLAST) [88,89]. Pairwise sequence similarity was calculated directly from BLAST output to assess evolutionary conservation across species.

### 4.2. Sampling and Characterization of Prepubertal Canine Reproductive Tissues

The here-used uterus, ovary, and uterine tubes were residual materials, provided by the Didactic Veterinary Hospital (OVUD) of the Department of Veterinary Sciences, University of Messina from eight prepubertal bitches (*Canis lupus familiaris*) that underwent ovariohysterectomy as part of routine clinical sterilization procedures at the owner’s request, and not specifically for research purposes. While ovariectomy is increasingly preferred in elective sterilization [90,91], these samples reflect OVUD’s standard clinical practice. To ensure that the selected animals had not undergone any heat cycles, authors have relied on a combination of qualifying criteria, including bitches owners’ declarations, age (between 4 and 6 months), and medical records, and a determinative criterion: the anatomical evaluation of the reproductive tract proving the absence of any ovarian activity indicative of previous estrous cycles, namely the presence of corpora lutea or large antral follicles. Histological and immunohistochemistry samples (four ovaries, four uteri, and four uterine tubes) were fixed in 4% paraformaldehyde, while Western blot analyses were conducted in the remaining samples stored at −80 °C.

### 4.3. Histology

Tissue samples were fixed in 4% paraformaldehyde in phosphate-buffered saline (PBS) (AAJ19943K2, Thermo Scientific, Waltham, MA, USA) 0.1 m (pH = 7.4) for 10 h, dehydrated through a graded ethanol series and then clarified in xylene for paraffin-wax embedding. Embedded tissue samples were then cut into 7 µm thick serial sections and collected on gelatin-coated microscope slides [22,23]. Sections were dried for 24 h, then processed for hematoxylin/eosin and Masson’s trichrome with Aniline Blue (Bio-optica Milano S.p.A, Milan, Italy, Cat. # 04–010802) staining.

### 4.4. Ion-Channel Expression and Primary Antibody Specificity in Canine Sample

#### 4.4.1. Alignment of Primary Antibodies Immunogen Sequences with the Respective Canine Proteins

In order to verify the specificity of the primary antibodies ASIC2, ASIC4, and PIEZO2 (for the antibodies’ details, see Table 2) to the canine proteins, an alignment was performed for the different antibody immunogen sequences and their respective sequences of canine proteins. The online software NCBI BLASTp (protein–protein BLAST https://www.ncbi.nlm.nih.gov/; accessed on 30 April 2025) was used for the peptide alignment as described before [88,89].

#### 4.4.2. Western Blot Analyses

To confirm the specificity of anti-ASIC2, ASIC4, and PIEZO2 (for the antibodies’ details, see Table 3) the Western blot using the same antibody was performed on three prepubertal bitches’ genital tracts’ (ovary, uterus, and uterine tubes) homogenates. Briefly, the samples were pooled and homogenized in radio-immunoprecipitation assay (RIPA) buffer (Sigma-Aldrich, St. Louis, MO, USA, Cat. # R0278) to which protease and phosphatase inhibitor cocktails were added (Sigma-Aldrich, St. Louis, MO, USA, Cat. # P8340; Sigma-Aldrich, St. Louis, MO, USA, Cat. # P5726; Sigma-Aldrich, St. Louis, MO, USA, Cat. # P0044). The lysates were analyzed by electrophoresis in polyacrylamide gel, and protein bands were then transferred to nitrocellulose membranes and blocked by immersion for three hours in phosphate-buffered saline containing 5% dry milk and 0.1% Tween-20. The nitrocellulose membrane, including the proteins, was then incubated in the primary antibodies (1:500) overnight at four degrees Celsius, followed by a two-hour incubation with a horseradish peroxidase-conjugated secondary Mouse anti-rabbit IgG-HRP (Santa Cruz Biotechnology, Santa Cruz, CA, USA, Cat. # Sc-2357) at room temperature. Finally, the reaction was revealed using a gel imaging system (ChemiScope 2850, Clinx Science Instruments Co., Ltd., Shanghai, China) as described previously [92].

### 4.5. Immunohistochemical and Immunofluorescent Detection of ASIC2, ASIC4, and PIEZO2 in Reproductive Tissues of Prepubertal Bitches

To analyze the expression of ASIC2, ASIC4 and PIEZO2 in the ovary, uterus, and uterine tubes of prepubertal bitches, serial sections were deparaffinized and rehydrated, washed in working buffer (Tris–HCl buffer 0.05 M, pH 7.5) containing 0.1% bovine serum albumin and 0.2% Triton-X 100, and later incubated in 0.3% H_2_O_2_ (PBS) solution for 3 min to prevent the activity of endogenous peroxidase. Then, the nonspecific binding was blocked by covering slides with 25% fetal bovine serum (F7524 Sigma-Aldrich) for 30 min to avoid non-specific binding. The incubation with ASIC2, ASIC4, and PIEZO2 rabbit polyclonal antibodies (see Table 3) was carried out overnight at four degrees Celsius in a humid chamber. Afterward, the sections were washed in the working buffer and incubated for an hour and a half at room temperature with a secondary antibody-peroxidase conjugate (see Table 3). The immunoreaction was visualized using 3-30-diaminobenzidine as a chromogen (DAB, Sigma-Aldrich, Inc., St. Louis, MO, USA Cat. #D5905). Some ovarian sections were counterstained with hematoxylin to contrast the oocytes’ immunopositivity. Finally, the slides were examined with photomicrographs were captured using the Leica Application Suite LAS V4.7, under a Leica DMRB light microscope equipped with a Leica MC 120 HD camera (Leica Microsystems GmbH, Wetzlar, Germany) [93,94]. Moreover, to improve the accuracy of the identification of the immunolabeled cells of the follicles, some serial sections have been processed for the immunofluorescence method. The sections were treated similarly to the immunohistochemistry protocol with a difference in the secondary antibody Anti-rabbit IgG (H + L) Alexa Fluor 594 (see Table 3), with which the sections were incubated (1 h, at room temperature in a dark humid chamber). Finally, the sections were washed and mounted using Fluoromount Aqueous Mounting Medium (Sigma-Aldrich, Inc., St. Louis, MO, USA. Cat. #F4680). A Zeiss LSMDUO confocal laser scanning microscope with META module (Carl Zeiss MicroImaging GmbH, Jena, Germany) was used to detect the immunofluorescence, and Zen 2011 (LSM 700 Zeiss software) was employed to process the images. Each image was rapidly acquired to minimize photodegradation.

Representative sections were incubated excluding the primary antibody as mentioned above to provide negative controls. In these circumstances, there was no evidence of positive immunostaining (Supplementary Material: Figure S4).

## 5. Conclusions

In conclusion, this study reports for the first time the expression of ASIC2, ASIC4, and PIEZO2 ion channels in the reproductive tracts of prepubertal bitches, as a first step toward understanding the implication of ion channels in bitches’ reproductive physiology. The detection of these channels in ovarian follicles, uterine tubes, uterine epithelium, and in ovarian and uterine arteries suggests their wide potential influence on bitches’ genital-tract physiology. These findings align with existing studies on other species, highlighting a possible across-species conservation of ion-channel roles in reproductive physiology.

Further investigations are needed to understand the mechanisms by which these channels influence reproductive processes, studying their differential expression in the other developmental stages.

## Figures and Tables

**Table 1:** A summary of sequence similarity metrics between the query proteins (ASIC2, ASIC4, and PIEZO2) and their homologs in six representative vertebrate species: *Canis lupus familiaris*, *Homo sapiens*, *Mus musculus*, *Anolis carolinensis*, *Gallus gallus*, and *Danio rerio*.

		Taxa	Max Score	Total Score	Query Cover	E-Value	Per. Ident	Acc. Len	Accession
Proteins	ASIC2	*Canis lupus familiaris*	-	-	-	-	-	-	XP_038533280.1
		*Homo sapiens*	790	790	95%	0.0	78.15%	563	NP_899233.1
		*Mus musculus*	1051	1051	100%	0.0	98.83%	512	NP_001029185.1
		*Anolis carolinensis*	918	918	100%	0.0	87.18%	521	XP_016850081.1
		*Gallus gallus*	775	775	96%	0.0	76.80%	507	XP_040548011.1
		*Danio rerio*	768	768	100%	0.0	71.67%	533	XP_068072559.1
	ASIC4	*Canis lupus* *familiaris*	-	-	-	-	-	-	XP_038303989.1
		*Homo sapiens*	1026	1026	100%	0.0	93.51%	520	NP_878267.3
		*Mus musculus*	1084	1084	100%	0.0	97.40%	539	NP_898843.1
		*Anolis carolinensis*	756	756	100%	0.0	70.61%	538	XP_008110128.2
		*Gallus gallus*	777	777	100%	0.0	73.11%	538	XP_015145601.3
		*Danio rerio*	692	692	100%	0.0	62.82%	539	NP_999952.1
	PIEZO2	*Canis lupus familiaris*	-	-	-	-	-	-	XP_038528610.1
		*Homo sapiens*	4942	4942	100%	0.0	90.24%	2752	AFC88283.1
		*Mus musculus*	4953	4953	100%	0.0	90.11%	2822	ADN28065.1
		*Anolis carolinensis*	4093	4093	100%	0.0	74.84%	2818	XP_062836599.1
		*Gallus gallus*	4427	4427	99%	0.0	79.72%	2853	XP_040520788.1
		*Danio rerio*	2045	3380	93%	0.0	62.89%	2972	XP_021323952.1

**Table 2:** A summary of the alignment results of anti-ASIC2, ASIC4, and PIEZO2 immunogens (from the human proteins) and their respective canine proteins. These data help assess the specificity of the antibodies based on sequence homology.

		Taxa	Query ID	Max Score	Total Score	Query Cover	E-Value	Per. Ident	Acc. Len	Accession
**Antibodies**	ASIC2	*Homo sapiens*	-	-	-	-	-	-	-	-
		*Canis lupus familiaris*	XP_038533280.1	67.4	67.4	6%	4 × 10^−20^	100.00%	32	Query_1685301
	ASIC4	*Homo sapiens*	-	-	-	-	-	-	-	-
		*Canis lupus familiaris*	XP_038303990.1	129	129	11%	6 × 10^−42^	98.33%	60	Query_481487
	PIEZO2	*Homo sapiens*	-	-	-	-	-	-	-	-
		*Canis lupus familiaris*	XP_048968903.1	85.5	85.5	1%	2 × 10^−25^	92.00%	50	Query_1375639

**Table 3:** Primary and secondary antibodies used in this study. Antibody dilutions were optimized for immunohistochemistry application.

Primary Antibodies				
ASIC2	Supplier: Invitrogen-Thermo Fisher Scientific, Waltham, MA, USA			
	Catalog number	Source	Dilution	Antibody ID
	OSR00098W	rabbit	1:100	AB_2220076
ASIC4	Supplier: Invitrogen-Thermo Fisher Scientific, Waltham, MA, USA			
	Catalog number	Source	Dilution	Antibody ID
	OSR00101W	rabbit	1:100	AB_2222665
PIEZO2	Supplier: Invitrogen-Thermo Fisher Scientific, Waltham, MA, USA			
	Catalog number	Source	Dilution	Antibody ID
	PA5-72976	rabbit	1:100	AB_2718830
**Secondary Antibodies**				
Anti-rabbit IgG-peroxidase conjugate	Supplier: Santa Cruz Biotechnology, Inc., Dallas, TX, USA			
	Catalog number	Source	Dilution	Antibody ID
	sc-2357	mouse	1:100	AB_628497
Anti-rabbit IgG (H + L) Alexa Fluor 594	Supplier: Molecular Probes, Invitrogen, Waltham, MA, USA			
	Catalog number	Source	Dilution	Antibody ID
	A32754	Donkey	1:300	AB_2762827

## Data Availability

All data presented in this study are available from the corresponding author upon responsible request.

## Supplementary Materials

The following supporting information can be downloaded at: https://www.mdpi.com/article/10.3390/ijms26094388/s1.

## References

[1] Concannon P.W. (2011). Reproductive Cycles of the Domestic Bitch. Anim. Reprod. Sci..

[2] Davis K.M., Partin A.M., Burghardt G.M., Springer C.M., Albright J.D. (2023). A Descriptive Methodology for Studying the Ontogeny of Object Play and Breed Differences in Dogs (*Canis lupus familiaris*). Animals.

[3] Gobello C. (2014). Prepubertal and Pubertal Canine Reproductive Studies: Conflicting Aspects. Reprod. Domest. Anim..

[4] Mo A., Dang Y., Wang J., Liu C., Yuan Y.C., Yang H. (2020). Sex Differences, Growth, Reproduction and Zinc Ion Homeostasis of Zebrafish after Chronic Dietary l-Selenomethionine Exposure. Chemosphere.

[5] Benko F., Urminská D., Ďuračka M., Tvrdá E. (2023). Signaling Roleplay between Ion Channels during Mammalian Sperm Capacitation. Biomedicines.

[6] Delgado-Bermúdez A., Yeste M., Bonet S., Pinart E. (2025). Physiological Role of Potassium Channels in Mammalian Germ Cell Differentiation, Maturation, and Capacitation. Andrology.

[7] Kim H.-J., Lee G.-S., Ji Y.-K., Choi K.-C., Jeung E.-B. (2006). Differential Expression of Uterine Calcium Transporter 1 and Plasma Membrane Ca^2+^ ATPase 1b during Rat Estrous Cycle. Am. J. Physiol. Endocrinol. Metab..

[8] Lee G.-S., Jeung E.-B. (2007). Uterine TRPV6 Expression during the Estrous Cycle and Pregnancy in a Mouse Model. Am. J. Physiol. Endocrinol. Metab..

[9] Strünker T., Goodwin N., Brenker C., Kashikar N.D., Weyand I., Seifert R., Kaupp U.B. (2011). The CatSper Channel Mediates Progesterone-Induced Ca^2+^ Influx in Human Sperm. Nature.

[10] Morales-Lázaro S.L., González-Ramírez R., Rosenbaum T. (2019). Molecular Interplay Between the Sigma-1 Receptor, Steroids, and Ion Channels. Front. Pharmacol..

[11] Díaz L., Ceja-Ochoa I., Restrepo-Angulo I., Larrea F., Avila-Chávez E., García-Becerra R., Borja-Cacho E., Barrera D., Ahumada E., Gariglio P. (2009). Estrogens and Human Papilloma Virus Oncogenes Regulate Human Ether-à-Go-Go-1 Potassium Channel Expression. Cancer Res..

[12] Irnaten M., Blanchard-Gutton N., Harvey B.J. (2008). Rapid Effects of 17β-Estradiol on Epithelial TRPV6 Ca^2+^ Channel in Human T84 Colonic Cells. Cell Calcium.

[13] O’Mahony F., Alzamora R., Betts V., LaPaix F., Carter D., Irnaten M., Harvey B.J. (2007). Female Gender-Specific Inhibition of KCNQ1 Channels and Chloride Secretion by 17β-Estradiol in Rat Distal Colonic Crypts. J. Biol. Chem..

[14] Alzamora R., O’Mahony F., Bustos V., Rapetti-Mauss R., Urbach V., Cid L.P., Sepúlveda F.V., Harvey B.J. (2011). Sexual Dimorphism and Oestrogen Regulation of KCNE3 Expression Modulates the Functional Properties of KCNQ1 K^+^ Channels. J. Physiol..

[15] Ghavideldarestani M., Butler A.E., Shirian S., Atkin S.L. (2019). Expression and Localization of Transient Receptor Potential Channels in the Bovine Uterus Epithelium throughout the Estrous Cycle. Mol. Biol. Rep..

[16] Ghavideldarestani M., Atkin S.L., Leese H.J., Sturmey R.G. (2016). Expression and Function of Transient Receptor Potential Channels in the Female Bovine Reproductive Tract. Theriogenology.

[17] Teilmann S.C., Byskov A.G., Pedersen P.A., Wheatley D.N., Pazour G.J., Christensen S.T. (2005). Localization of Transient Receptor Potential Ion Channels in Primary and Motile Cilia of the Female Murine Reproductive Organs. Mol. Reprod. Dev..

[18] Boscardin E., Alijevic O., Hummler E., Frateschi S., Kellenberger S. (2016). The Function and Regulation of Acid-Sensing Ion Channels (ASICs) and the Epithelial Na^+^ Channel (ENaC): IUPHAR Review 19. Br. J. Pharmacol..

[19] Gründer S., Pusch M. (2015). Biophysical Properties of Acid-Sensing Ion Channels (ASICs). Neuropharmacology.

[20] Montalbano G., Levanti M., Mhalhel K., Abbate F., Laurà R., Guerrera M.C., Aragona M., Germanà A. (2021). Acid-Sensing Ion Channels in Zebrafish. Animals.

[21] Cheng Y.-R., Jiang B.-Y., Chen C.-C. (2018). Acid-Sensing Ion Channels: Dual Function Proteins for Chemo-Sensing and Mechano-Sensing. J. Biomed. Sci..

[22] Hanukoglu I. (2017). ASIC and ENaC Type Sodium Channels: Conformational States and the Structures of the Ion Selectivity Filters. FEBS J..

[23] Wu J., Lewis A.H., Grandl J. (2017). Touch, Tension, and Transduction—The Function and Regulation of Piezo Ion Channels. Trends Biochem. Sci..

[24] Coste B., Mathur J., Schmidt M., Earley T.J., Ranade S., Petrus M.J., Dubin A.E., Patapoutian A. (2010). Piezo1 and Piezo2 Are Essential Components of Distinct Mechanically Activated Cation Channels. Science.

[25] Ranade S.S., Qiu Z., Woo S.-H., Hur S.S., Murthy S.E., Cahalan S.M., Xu J., Mathur J., Bandell M., Coste B. (2014). Piezo1, a Mechanically Activated Ion Channel, Is Required for Vascular Development in Mice. Proc. Natl. Acad. Sci. USA.

[26] Young M., Lewis A.H., Grandl J. (2022). Physics of Mechanotransduction by Piezo Ion Channels. J. Gen. Physiol..

[27] Parpaite T., Coste B. (2017). Piezo Channels. Curr. Biol..

[28] Aragona M., Mhalhel K., Cometa M., Franco G.A., Montalbano G., Guerrera M.C., Levanti M., Laurà R., Abbate F., Vega J.A. (2024). Piezo 1 and Piezo 2 in the Chemosensory Organs of Zebrafish (*Danio rerio*). Int. J. Mol. Sci..

[29] Aragona M., Mhalhel K., Pansera L., Montalbano G., Guerrera M.C., Levanti M., Laurà R., Abbate F., Vega J.A., Germanà A. (2024). Localization of Piezo 1 and Piezo 2 in Lateral Line System and Inner Ear of Zebrafish (*Danio rerio*). Int. J. Mol. Sci..

[30] Butenas A.L.E., Rollins K.S., Parr S.K., Hammond S.T., Ade C.J., Hageman K.S., Musch T.I., Copp S.W. (2022). Novel Mechanosensory Role for Acid Sensing Ion Channel Subtype 1a in Evoking the Exercise Pressor Reflex in Rats with Heart Failure. J. Physiol..

[31] Evans E.L., Cuthbertson K., Endesh N., Rode B., Blythe N.M., Hyman A.J., Hall S.J., Gaunt H.J., Ludlow M.J., Foster R. (2018). Yoda1 Analogue (Dooku1) Which Antagonizes Yoda1-Evoked Activation of Piezo1 and Aortic Relaxation. Br. J. Pharmacol..

[32] Akanji O., Weinzierl N., Schubert R., Schilling L. (2019). Acid Sensing Ion Channels in Rat Cerebral Arteries: Probing the Expression Pattern and Vasomotor Activity. Life Sci..

[33] Arishe O.O., McKenzie J., Dela Justina V., Dos Anjos Moraes R., Webb R.C., Priviero F. (2023). Piezo1 Channels Mediate Vasorelaxation of Uterine Arteries from Pseudopregnant Rats. Front. Physiol..

[34] Alcaino C., Farrugia G., Beyder A. (2017). Mechanosensitive Piezo Channels in the Gastrointestinal Tract. Curr. Top. Membr..

[35] Stewart T.A., Davis F.M. (2019). Formation and Function of Mammalian Epithelia: Roles for Mechanosensitive PIEZO1 Ion Channels. Front. Cell Dev. Biol..

[36] Kikuchi S., Ninomiya T., Kawamata T., Tatsumi H. (2008). Expression of ASIC2 in Ciliated Cells and Stereociliated Cells. Cell Tissue Res..

[37] Aspinall V. (2011). Reproductive System of the Dog and Cat—Part 3. Reproductive Physiology of the Bitch. Vet. Nurs. J..

[38] Linde-Forsberg C. (2001). Biology of Reproduction and Modern Reproductive Technology. The Genetics of the Dog.

[39] Nagashima J.B., Songsasen N. (2021). Canid Reproductive Biology: Norm and Unique Aspects in Strategies and Mechanisms. Animals.

[40] Carvacho I., Piesche M., Maier T.J., Machaca K. (2018). Ion Channel Function During Oocyte Maturation and Fertilization. Front. Cell Dev. Biol..

[41] Bronson F.H. (1985). Mammalian Reproduction: An Ecological Perspective. Biol. Reprod..

[42] Jegla T.J., Zmasek C.M., Batalov S., Nayak S.K. (2009). Evolution of the Human Ion Channel Set. Comb. Chem. High Throughput Screen.

[43] Pascual-García A., Abia D., Méndez R., Nido G.S., Bastolla U. (2010). Quantifying the Evolutionary Divergence of Protein Structures: The Role of Function Change and Function Conservation. Proteins Struct. Funct. Bioinform..

[44] Meinel T. (2009). Function and Homology of Proteins Similar in Sequence: Phylogenetic Profiling. Handbook of Research on Systems Biology Applications in Medicine.

[45] Steinhauer N., Boos A., Günzel-Apel A.-R. (2004). Morphological Changes and Proliferative Activity in the Oviductal Epithelium during Hormonally Defined Stages of the Oestrous Cycle in the Bitch. Reprod. Domest. Anim..

[46] Groppetti D., Aralla M., Bronzo V., Bosi G., Pecile A., Arrighi S. (2015). Periovulatory Time in the Bitch: What’s New to Know?: Comparison between Ovarian Histology and Clinical Features. Anim. Reprod. Sci..

[47] Schäfer-Somi S., Deichsel K., Beceriklisoy H., Korkmaz D., Walter I., Aslan S. (2017). Morphological, Histological and Molecular Investigations on Canine Uterine Tissue after Ovariectomy. Theriogenology.

[48] Solano-Gallego L., Masserdotti C., Raskin R.E., Meyer D.J. (2015). Reproductive System. Canine Feline Cytology.

[49] Eppig J.J. (2001). Oocyte Control of Ovarian Follicular Development and Function in Mammals. Reproduction.

[50] Fortune J.E., Cushman R.A., Wahl C.M., Kito S. (2000). The Primordial to Primary Follicle Transition. Mol. Cell. Endocrinol..

[51] Gougeon A. (1996). Regulation of Ovarian Follicular Development in Primates: Facts and Hypotheses. Endocr. Rev..

[52] Mhalhel K., Arena R., Rizzo M., Piccione G., Aragona M., Levanti M., Aragona F., Arfuso F. (2024). Potential Implications of Acid-Sensing Ion Channels ASIC2 and ASIC4 in Gonadal Differentiation of Dicentrarchus Labrax Subjected to Water Temperature Increase during Gonadal Development. Animals.

[53] Wang G., Wang Y.-Z., Yu Y., Wang J.-J. (2019). Inhibitory ASIC2-Mediated Calcineurin/NFAT against Colorectal Cancer by Triterpenoids Extracted from Rhus Chinensis Mill. J. Ethnopharmacol..

[54] Zhou Z.-H., Song J.-W., Li W., Liu X., Cao L., Wan L.-M., Tan Y.-X., Ji S.-P., Liang Y.-M., Gong F. (2017). The Acid-Sensing Ion Channel, ASIC2, Promotes Invasion and Metastasis of Colorectal Cancer under Acidosis by Activating the Calcineurin/NFAT1 Axis. J. Exp. Clin. Cancer Res..

[55] Tosti E. (2006). Calcium Ion Currents Mediating Oocyte Maturation Events. Reprod. Biol. Endocrinol..

[56] Joshi T., Wilhite A., Chokshi S., Singleton M.H., Scalici J., Lee K. (2024). Abstract A032: The Role of ASIC2 and Calcium Influx in Epithelial Ovarian Cancer Pathogenesis. Cancer Res..

[57] Nader N., Kulkarni R.P., Dib M., Machaca K. (2013). How to Make a Good Egg!: The Need for Remodeling of Oocyte Ca^2+^ Signaling to Mediate the Egg-to-Embryo Transition. Cell Calcium.

[58] Kishimoto T. (2015). Entry into Mitosis: A Solution to the Decades-Long Enigma of MPF. Chromosoma.

[59] Miao Y.-L., Stein P., Jefferson W.N., Padilla-Banks E., Williams C.J. (2012). Calcium Influx-Mediated Signaling Is Required for Complete Mouse Egg Activation. Proc. Natl. Acad. Sci. USA.

[60] Wakai T., Mehregan A., Fissore R.A. (2019). Ca^2+^ Signaling and Homeostasis in Mammalian Oocytes and Eggs. Cold Spring Harb. Perspect. Biol..

[61] Stricker S.A. (1999). Comparative Biology of Calcium Signaling during Fertilization and Egg Activation in Animals. Dev. Biol..

[62] Tokmakov A.A., Stefanov V.E., Iwasaki T., Sato K.-I., Fukami Y. (2014). Calcium Signaling and Meiotic Exit at Fertilization in Xenopus Egg. Int. J. Mol. Sci..

[63] Webb S.E., Miller A.L. (2013). Ca^2+^ Signaling during Activation and Fertilization in the Eggs of Teleost Fish. Cell Calcium.

[64] Bai X., Bouffard J., Lord A., Brugman K., Sternberg P.W., Cram E.J., Golden A. (2020). Caenorhabditis Elegans PIEZO Channel Coordinates Multiple Reproductive Tissues to Govern Ovulation. eLife.

[65] Douguet D., Honoré E. (2019). Mammalian Mechanoelectrical Transduction: Structure and Function of Force-Gated Ion Channels. Cell.

[66] Li X., Hu J., Zhao X., Li J., Chen Y. (2022). Piezo Channels in the Urinary System. Exp. Mol. Med..

[67] Gudipaty S.A., Lindblom J., Loftus P.D., Redd M.J., Edes K., Davey C.F., Krishnegowda V., Rosenblatt J. (2017). Mechanical Stretch Triggers Rapid Epithelial Cell Division through Piezo1. Nature.

[68] Mim M.S., Kumar N., Levis M., Unger M.F., Miranda G., Gazzo D., Robinett T., Zartman J.J. (2024). Piezo Regulates Epithelial Topology and Promotes Precision in Organ Size Control. Cell Rep..

[69] Piddini E. (2017). Epithelial Homeostasis: A Piezo of the Puzzle. Curr. Biol..

[70] Enders A.C., Given R.L., Schlafke S. (1978). Differentiation and Migration of Endoderm in the Rat and Mouse at Implantation. Anat. Rec..

[71] Bae C., Sachs F., Gottlieb P.A. (2011). The Mechanosensitive Ion Channel Piezo1 Is Inhibited by the Peptide GsMTx4. Biochemistry.

[72] Hennes A., Held K., Boretto M., De Clercq K., Van den Eynde C., Vanhie A., Van Ranst N., Benoit M., Luyten C., Peeraer K. (2019). Functional Expression of the Mechanosensitive PIEZO1 Channel in Primary Endometrial Epithelial Cells and Endometrial Organoids. Sci. Rep..

[73] Mahapatra C., Kumar R. (2024). Biophysical Mechanisms of Vaginal Smooth Muscle Contraction: The Role of the Membrane Potential and Ion Channels. Pathophysiology.

[74] Suarez S.S., Wolfner M.F. (2021). Cilia Take the Egg on a Magic Carpet Ride. Proc. Natl. Acad. Sci. USA.

[75] Yuan S., Wang Z., Peng H., Ward S.M., Hennig G.W., Zheng H., Yan W. (2021). Oviductal Motile Cilia Are Essential for Oocyte Pickup but Dispensable for Sperm and Embryo Transport. Proc. Natl. Acad. Sci. USA.

[76] Arishe O.O., Ebeigbe A.B., Webb R.C. (2020). Mechanotransduction and Uterine Blood Flow in Preeclampsia: The Role of Mechanosensing Piezo 1 Ion Channels. Am. J. Hypertens..

[77] Rode B., Shi J., Endesh N., Drinkhill M.J., Webster P.J., Lotteau S.J., Bailey M.A., Yuldasheva N.Y., Ludlow M.J., Cubbon R.M. (2017). Piezo1 Channels Sense Whole Body Physical Activity to Reset Cardiovascular Homeostasis and Enhance Performance. Nat. Commun..

[78] Barnett S.D., Asif H., Buxton I.L.O. (2023). Novel Identification and Modulation of the Mechanosensitive Piezo1 Channel in Human Myometrium. J. Physiol..

[79] Zhang Y., Hermanson M.E., Eddinger T.J. (2013). Tonic and Phasic Smooth Muscle Contraction Is Not Regulated by the PKCα—CPI-17 Pathway in Swine Stomach Antrum and Fundus. PLoS ONE.

[80] Brading A.F. (2006). Spontaneous Activity of Lower Urinary Tract Smooth Muscles: Correlation between Ion Channels and Tissue Function. J. Physiol..

[81] Amberg G.C., Koh S.D., Imaizumi Y., Ohya S., Sanders K.M. (2003). A-Type Potassium Currents in Smooth Muscle. Am. J. Physiol. Cell Physiol..

[82] Jackson W.F., Khalil R.A. (2017). Chapter Three—Potassium Channels in Regulation of Vascular Smooth Muscle Contraction and Growth. Advances in Pharmacology.

[83] Pablo J.L., DeCaen P.G., Clapham D.E. (2017). Progress in Ciliary Ion Channel Physiology. J. Gen. Physiol..

[84] Enuka Y., Hanukoglu I., Edelheit O., Vaknine H., Hanukoglu A. (2012). Epithelial Sodium Channels (ENaC) Are Uniformly Distributed on Motile Cilia in the Oviduct and the Respiratory Airways. Histochem. Cell Biol..

[85] Chapleau M.W., Lu Y., Abboud F.M., Hamill O.P. (2007). Chapter 19—Mechanosensitive Ion Channels in Blood Pressure-Sensing Baroreceptor Neurons. Current Topics in Membranes.

[86] López-Ramírez O., González-Garrido A. (2023). The Role of Acid Sensing Ion Channels in the Cardiovascular Function. Front. Physiol..

[87] Lu Y., Ma X., Sabharwal R., Snitsarev V., Morgan D., Rahmouni K., Drummond H.A., Whiteis C.A., Costa V., Price M. (2009). The Ion Channel ASIC2 Is Required for Baroreceptor and Autonomic Control of the Circulation. Neuron.

[88] Mhalhel K., Briglia M., Aragona M., Porcino C., Abbate F., Guerrera M.C., Laurà R., Krichen Y., Guerbej H., Germanà A. (2023). Nothobranchius as a Model for Anorexia of Aging Research: An Evolutionary, Anatomical, Histological, Immunohistochemical, and Molecular Study. Ann. Anat. Anat. Anz..

[89] Mhalhel K., Montalbano G., Giurdanella G., Abbate F., Laurà R., Guerrera M.C., Germanà A., Levanti M. (2022). Histological and Immunohistochemical Study of Gilthead Seabream Tongue from the Early Stage of Development: TRPV4 Potential Roles. Ann. Anat..

[90] DeTora M., McCarthy R.J. (2011). Ovariohysterectomy versus Ovariectomy for Elective Sterilization of Female Dogs and Cats: Is Removal of the Uterus Necessary?. J. Am. Vet. Med. Assoc..

[91] Romagnoli S., Krekeler N., de Cramer K., Kutzler M., McCarthy R., Schaefer-Somi S. (2024). WSAVA Guidelines for the Control of Reproduction in Dogs and Cats. J. Small Anim. Pract..

[92] Germanà A., Guerrera M.C., Laurà R., Levanti M., Aragona M., Mhalhel K., Germanà G., Montalbano G., Abbate F. (2020). Expression and Localization of BDNF/TrkB System in the Zebrafish Inner Ear. Int. J. Mol. Sci..

[93] Aragona M., Porcino C., Guerrera M.C., Montalbano G., Laurà R., Levanti M., Abbate F., Cobo T., Capitelli G., Calapai F. (2022). Localization of BDNF and Calretinin in Olfactory Epithelium and Taste Buds of Zebrafish (*Danio rerio*). Int. J. Mol. Sci..

[94] Mhalhel K., Kadmi Y., Ben Chira A., Levanti M., Pansera L., Cometa M., Sicari M., Germanà A., Aragona M., Montalbano G. (2024). *Urtica dioica* Extract Abrogates Chlorpyrifos-Induced Toxicity in Zebrafish Larvae. Int. J. Mol. Sci..

