# Supplementary material for: Ion-Channel Proteins in the Prepubertal Bitch Reproductive System: The Immunolocalization of ASIC2, ASIC4, and PIEZO2
Source: Int J Mol Sci. 2025 May 5;26(9):4388. doi: 10.3390/ijms26094388 (PMC12072602; doi:10.3390/ijms26094388)
Supplement: Supplementary file 1 [file ijms-26-04388-s001.zip › ijms-3482212-supplementary.pdf]

```
NP 001029185.1   1        MDL-KESPSEGSL----QPS   SIQ   ----IFANTSTLHGIRHIFVYGPLTI    --RRVLWAVAFVGSLGLL   56
XP 016850081.1   1     [ 6]MDL-KESISEGSLGSL-QPS   SLQ   ----IFANTSTLHGIRHIFVYGPMTV    --RRALWALVFVASLGIL   65
NP 899233.1      1     [19]FRMaREEPAPAALAAAgQPG[7]ALQ[5]RRGRPSLSRAKLHGLRHMCAGRTAAG[2]FQRRALWVLAFCTSFGLL   100
XP 040548011.1   1        --------------------   ---[2]RGRRVPALRTRLHGLRHMCSRRAA--    LPRRALWVLAFCTALGLL   44
NP 999953.1      1        MDL-KE--ACGSEASReTES[4]SLG[2]SSWQSFAHRSTLHGLRFIFPYSSSSS[7]TSRRLLWSAALLASLVLL   77

NP 001029185.1   57    LVESSERVSYYFSYQHVTKVDEVVAQSLVFPAVTLCNLNGFRFSRLTTNDLYHAGELLALLDVNLQIPDPHLADPTVLEA   136
XP 016850081.1   66    LVESSERVVYYFSYQHVTKVDEVVANNLVFPAVTICNLNGFRFSRLTTNDLYHAGDLLALLDVNLQILHPHLADPAVLAI   145
NP 899233.1      101   LSWSSNRLLYWLSFPSHTRVHREWSRQLPFPAVTVCNNNPLRFPRLSKGDLYYAGHWLGLLLPN------RTARPLVSEL   174
XP 040548011.1   45    LSWSSNRLLHWLSFPSHTQVRTEWSRQLAFPAVTLCNNNPLRFPRLSKGDLYYAGHWLGLLLPN------RTARPLLTEL   118
NP 999953.1      78    VLESTERLAYFLSYPHVTSVDAVVSGSLVFPAVTVCNLNAYRFTRLTQNDLYHAGELLALLDVHLQIPEPHLAEPHVLAF   157

NP 001029185.1   137   LR     QKANFKHYKPKQFS---MLEFLHRVGHDLKDMMLYCKFKGQECGHQDFTTVFTKYGKCYMFNSGEDGKPLLTTV   209
XP 016850081.1   146   LQ     EKTNFQHHRAKVFS---MSEFLGRVGHDMKEMLLYCKFRGQECSHKNFTTVFTKYGKCYMFNSGEDGHPLLTTF   218
NP 899233.1      175   LR[10]KLADFRLFLPPRHFegiSAAFMDRLGHQLEDMLLSCKYRGELCGPHNFSSVFTKYGKCYMFNSGEDGKPLLTTV   260
XP 040548011.1   119   LR[10]KLADFRLFLPPRHYegiSADFMDRLGHQLEDMLLSCKYRGELCGPHNFSAVFTKYGKCYMFNSGEEGRPLLTTV   204
NP 999953.1      158   LT     EKSNFTNYRPKPFS---MREFTERVGHDLKEMMLYCRFQGQECSHQDFKTVFTRYGKCYMFNAAEEGKTLRTTM   230

NP 001029185.1   210   KGGTGNGLEIMLDIQQDEYLPIWGETEETTFEAGVKVQIHSQSEPPFIQELGFGVAPGFQTFVATQEQRLTYLPPPWGEC   289
XP 016850081.1   219   KGGTGNGLEIMLDIQQDEYLPIWGETEETTFEAGVKVQIHSQSEPPFVQELGFGVAPGFQTFVATQEQRLTYLPPPWGEC   298
NP 899233.1      261   KGGTGNGLEIMLDIQQDEYLPIWGETEETTFEAGVKVQIHSQSEPPFIQELGFGVAPGFQTFVATQEQRLTYLPPPWGEC   340
XP 040548011.1   205   KGGTGNGLEIMLDIQQDEYLPIWGETEETTFEAGVKVQIHSQSEPPFVQELGFGVAPGFQTFVATQEQRLTYLPPPWGEC   284
NP 999953.1      231   KGGTGNGLEIMLDIQQDEYLPVWGETEETAFEAGVRVQIHSQAEPPFVHELGFGVAPGFQTFVATQEQRLTYLPPPWGEC   310

NP 001029185.1   290   RSSEMGLDFFPVYSITACRIDCETRYIVENCNCRMVHMPGDAPFCTPEQHKECAEPALGLLAEKDSNYCLCRTPCNLTRY   369
XP 016850081.1   299   RSSDFGLDFFPVYSITACRIDCETRYVVENCNCKMVHMPGDAPFCTPEQYKECAEPALVVLGEKDSGYCVCQMPCNLTRY   378
NP 899233.1      341   RSSEMGLDFFPVYSITACRIDCETRYIVENCNCRMVHMPGDAPFCTPEQHKECAEPALGLLAEKDSNYCLCRTPCNLTRY   420
XP 040548011.1   285   RSSDMGLDFFPVYSITACRIDCETRYIVENCNCKMVHMPGDAPFCTPEQYKECAEPALGLLAEKDSNYCICRTPCNLTRY   364
NP 999953.1      311   VSRALDSGLFQVYSVSACRIECETRYIVENCNCRMVYMPGDSPYCTPEQYKDCAEPALAALSAVEGTNCICRSPCNMTRY   390

NP 001029185.1   370   NKELSMVKIPSKTSAKYLEKKFNKSEKYISENILVLDIFFEALNYETIEQKKAYEVAALLGDIGGQMGLFIGASILTILE   449
XP 016850081.1   379   NKELSMVKIPSKTSAKYLEKKFNKSEKYISENILVLDIFFEALNYETIEQKKAYEVAALLGDIGGQMGLFIGASILTILE   458
NP 899233.1      421   NKELSMVKIPSKTSAKYLEKKFNKSEKYISENILVLDIFFEALNYETIEQKKAYEVAALLGDIGGQMGLFIGASILTILE   500
XP 040548011.1   365   NKELSMVKIPSKTSAKYLEKKFNKSEKYISENILVLDIFFEALNYETIEQKKAYEVAALLGDIGGQMGLFIGASILTILE   444
NP 999953.1      391   NKELSMVKIPSKTSARYLEKKFNRSEKYITDNILVLDVFFEALNYETIEQKKAYEVAGLLGDIGGQMGLFIGASILTLLE   470

NP 001029185.1   450   LFDYIYELIKEKLLDLLGKEEEEGSHDENMSTCDTMPNHSETISHTVNVPLQTALGTLEEIAC   512
XP 016850081.1   459   IFDYIYELLKEKLLDLLGQEEEEGSHDENVSTCEPLPNHSDPISHTVTVPLQTTLGTLEEIAC   521
NP 899233.1      501   LFDYIYELIKEKLLDLLGKEEDEGSHDENVSTCDTMPNHSETISHTVNVPLQTTLGTLEEIAC   563
XP 040548011.1   445   LFDYIYELIKEKLLDLLGKEEEEGSHDENVSTCDPMPNHSETISHTVNVPLQATLGTLEEIAC   507
NP 999953.1      471   LFDYAYEVVKERLLDLLNREEEEESHGEDVSTCDPVVNHSESISHTVSVPLQTTLGTLEEIAC   533
```

Figure S1: Multiple Alignment of Dog (XP_038533280.1), Human (NP_899233.1), Mice (NP_001029185.1), chicken (XP_040548011.1), Zebrafish (NP_999953.1) and Green anole (XP_016850081.1) Asic2 result.

```
NP_898843.1      1    MPIEIVCKIKFAEEDAKPKEKEAGDEQSLLgaaQGPAAP--RDLATFASTSTLHGLGRACGPGPHGLRRTLWALALLTSL  78
NP_878267.3      1    MPIEIVCKIKFAEEDAKPKEKEAGDEQSLLgavAPGAAP--RDLATFASTSTLHGLGRACGPGPHGLRRTLWALALLTSL  78
XP_015145601.3   1    MPIEIVCKIKFAEEDEKQKEKEEGDKESLI---EEPARPRSRDLAAFASTSTLHGLGHICGAGRPGVRQTLWALAFLASL  77
XP_008110128.2   1    MPIEIVCKIKFAEEDEKQAAKEEGDKESLL---EEPRRPHRRDLAAFAGASTLHGFGHICRSGRLGVRQALWALAFAASL  77
NP_999952.1      1    MPIEFVCKIKFAEEDEKQKGNQDGDKESLI---EESCSPPTKDLAGFASASSLHGINHIFVSGRLGVRQTLWALAFLVSL  77

NP_898843.1      79   AAFLYQAASLARGYLTRPHLVAMDPAAPAPVAgFPAVTLCNINRFRHSALSDADIFHLANLTGLPPKDRDGHRAAGLRYP  158
NP_878267.3      79   AAFLYQAAGLARGYLTRPHLVAMDPAAPAPVAgFPAVTLCNINRFRHSALSDADIFHLANLTGLPPKDRDGHRAAGLRYP  158
XP_015145601.3   78   AFFLYQAAKSALLYLEHPHVVALDEEAIREMV-FPAITICNINRYRHSALTDADIFHLANMTGLPPKDRDGHKATDLLYP  156
XP_008110128.2   78   AFFLYQAARSALFYLEHPHVTTLDEEATQRMV-FPAVTICNINRFRHSALTDADIFHLANMTGLPPKDRDGHRASDLLYP  156
NP_999952.1      78   ALFLYQAAKCAISYLEHPHVTALNEEATPEMV-FPAVTICNINRFRFSALTDADIYHLANLTGLPPKNKDGHKPTDLEYP  156

NP_898843.1      159  EPDMVDILNRTGHQLADMLKSCNFSGHHCSASNFSVVYTRYGKCYTFNADPQSSLPSRAGGMGSGLEIMLDIQQEEYLPI  238
NP_878267.3      159  EPDMVDILNRTGHQLADMLKSCNFSGHHCSASNFSVVYTRYGKCYTFNADPRSSLPSRAGGMGSGLEIMLDIQQEEYLPI  238
XP_015145601.3   157  DPDMADIVNRTGHQLDDMLKSCNFSGENCSSRDFMVVYTRYGKCYTFNGDRRNPRVTRQGGMGNGLEIMLDIQQEEYLPI  236
XP_008110128.2   157  DPDMADIVNRTGHQLDDMLKNCSFSGENCSAENFTVVYTRYGKCYTFNGNRNKPRVTRQSGMGNGLEIMLDIQQEEYLPI  236
NP_999952.1      157  APDMQDIFNRTGHQLEEMLKSCNFSGQNCSAEDFTVVYTRYGKCYTFNGNKTTSRKTKQGGMGNGLEIMLDIQQDDYLPI  236

NP_898843.1      239  WRETNETSFEAGIRVQIHSQEEPPYIHQLGFGVSPGFQTFVSCQEQRLTYLPQPWGNCRAESElREPELQGYSAYSVSAC  318
NP_878267.3      239  WRETNETSFEAGIRVQIHSQEEPPYIHQLGFGVSPGFQTFVSCQEQRLTYLPQPWGNCRAESElREPELQGYSAYSVSAC  318
XP_015145601.3   237  WRETNETSFEAGIRVQIHSQDEPPYIHQLGFGVSPGFQTFVSCQEQRLTYLPQPWGNCRASVQ-GEQMLPGYDTYSIAAC  315
XP_008110128.2   237  WRETNETSFEAGIRVQIHSQDEPPYIHQLGFGVSPGFQTFVSCQEQRLTYLPQPWGSCRASVK-GEQVLPGYESYSIAAC  315
NP_999952.1      237  WKETNETSLEAGIRVQIHSQDEPPYIHQLGFGVSPGFQTFVSCQEQRLTYLPQPWGNCRSTS---EQMIPGYDTYSISAC  313

NP_898843.1      319  RLRCEKEAVLQRCHCRMVHMPGNETICPPNIYIECADHTLDSLGGGSEGPCFCPTPCNLTRYGKEISMVKIPNRGSARYL  398
NP_878267.3      319  RLRCEKEAVLQRCHCRMVHMP-------------------DSLGGGPEGPCFCPTPCNLTRYGKEISMVRIPNRGSARYL  379
XP_015145601.3   316  RLQCEKEAVVRSCHCRMVHMPGNESICSPNVYIECADHTLDAAVEDSQERCSCPTPCNLTRYGKEISMVRIPNKGSARYL  395
XP_008110128.2   316  RLQCEKEAVVRNCQCRMVHMPGNETICSPNVYIECADHTLDTAVEDSQDRCVCPTPCNMTRYGKEISMVRIPNKGSARYL  395
NP_999952.1      314  RLRCETLEVLRECKCRMVHMPGDANICTPS-DIKCVDKALALLQKSSGDTCFCETPCNLTRYGKELSMVKIPSKGSARYL  392

NP_898843.1      399  ARKYNRNETYIRENFLVLDVFFEALTSEAMEQQAAYGLSALLGDLGGQMGLFIGASILTLLEILDYIYEVSWDRLKRVWR  478
NP_878267.3      380  ARKYNRNETYIRENFLVLDVFFEALTSEAMEQRAAYGLSALLGDLGGQMGLFIGASILTLLEILDYIYEVSWDRLKRVWR  459
XP_015145601.3   396  ARKYNKNETYIRENFLVLDIFFEALNYEAIEQKKAYDLAGLLGDIGGQMGLFIGASILTILEILDYIYEVIRDRVSRVLR  475
XP_008110128.2   396  ARKYNRNETYIRDNFLVLDIFFEALNYESIEQKKAYNLAGLLGDIGGQMGLFIGASVLTVLEILDYIYEVIRDKVAHGLR  475
NP_999952.1      393  SRKYDKSEDYIRDNFLVLDIFFEALNYETIEQKKAYDVAGLLGDIGGQMGLFIGASVLTILEILDYVYEVIKHRLERLLR  472

NP_898843.1      479  RPKTPLRTS   TGGISTLGLQELKEQSPCPSRGR-AEGGGASSLLPNHHHPHG-PPGSLFEDFAC  539
NP_878267.3      460  RPKTPLRTS   TGGISTLGLQELKEQSPCPSRGR-VEGGGVSSLLPNHHHPHG-PPGGLFEDFAC  520
XP_015145601.3   476  RSKPPLKKP   SGSIATLGLEELKDQSPCETLGRHVEGTYNAGILPNHHHRHHyPHQGVFEDFAC  538
XP_008110128.2   476  RSKPALKKP   SGSIATLGLEEIKEQSPCDTLGRHAEGTYPTGLLPNHHHRHHySAQGVFEDFAC  538
NP_999952.1      473  PQRDDKKQT[5]ASTVATVNLEEMKAKDSSEMSRSHSEGAYANTILPNHHHHHR-THHRVSEDFAC  539
```

Figure S2: Multiple Alignment of Canine (XP_038303989.1), humans (NP_878267.3), mice (NP_898843.1), green anole (XP_008110128.2), chicken (XP_015145601.3) and zebrafish (NP_999952.1) Asic4 result.

```
ADN28065.1       1     MA    SEVVCGLIFRLLLPICLAVACAFRYNGLSFVYLIYLLLIPLFSEPTKATMQGHTGRLLQSLCITSLSFLLLHIIF  77
AFC88283.1       1     MA    SEVVCGLIFRLLLPICLAVACAFRYNGLSFVYLIYLLLIPLFSEPTKTTMQGHTGRLLKSLCFISLSFLLLHIIF  77
XP_040520788.1   1     MS[7]QEERCGRT----PGSTERRACAFRYNGLSFVYLIYLLLIPLFSEPTKTTMQGHTGRLLKSLCFTSLSFLLLHIIF  80
XP_062836599.1   1     MA    TEVVCGLIFRLLLPICLVTACAFRYNGLSFVYLIYLLLIPLFSEPTKTTMQGHTGRLLKSLCFMSLTFLLLHIIF  77
XP_021323952.1   1     MA    SEVVCGLVFRVLLPVCLAAACLFRFNGLSFIYLLFLLLIPLFPEPSSITMRGQTGYLLKSLCCCSVTFLFFHIIY  77

ADN28065.1       78    HITLASLEAQHRITPAYNCSTWEKTFRQIGFESLKGADAGNGIRVFVPDIGMFIASLTIWLVCRTIVKKPDTEEIAQLNS  157
AFC88283.1       78    HITLVSLEAQHRIAPGYNCSTWEKTFRQIGFESLKGADAGNGIRVFVPDIGMFIASLTIWLLCRNIVQKPVTDEAAQSNP  157
XP_040520788.1   81    QITINSLEAGRTIEPGFNCSTWEKTLRQIGFESVKGADAGNVIRLFVPDIGMFIASLTIWLLCRNMVQKAINEDAVHCNA  160
XP_062836599.1   78    QITVNSLEAANTIEPDYNCSTWEKILRQIGFESVKGADVGNGIRIFVPDIGMFIASLTIWLICRKIVEKTAEEDAGLYNA  157
XP_021323952.1   78    QITVHSLLARGRVDSGFNCSVWEKSIRQIGFESVIGADAGNGIRVFLPDIGMFMASLGVWLLCRKLLHQRPTEDMAQDNQ  157

ADN28065.1       158   ECENEELAG-GEKMDS     EEALIYEEDLD   GEEGM    EGELEESTKLKILRRFASVASKLKEFIGNMITTAGKV  225
AFC88283.1       158   EFENEELAE-GEKIDS     EEALIYEEDFN   GGDGV    EGELEESTKLKMFRRLASVASKLKEFIGNMITTAGKV  225
XP_040520788.1   161   QFENEEMAV-REKLEP     DDALMYEEDLD   DDCG-    EAEFEETMKLKLLRRIASLASKLREFIGNMIITTGKV  227
XP_062836599.1   158   QFENEDMNE-AERLEA     EDALICE-DLD   DECG-    EEEFEETTKLKLLRRIASIASKLKEIIGNIITTAGKV  223
XP_021323952.1   158   QFLSEEKEEeNEKLEN[11]DDEMLFDEDFD[6]EEDGE[13]EEEVQESTKMKILRAVGGVAAKLKEIIGNLITTAGQV  256

ADN28065.1       226   VVTILLGSSGMMLPSLTSAVYFFVFLGLCTWWSWCRTFDPLLFGCLCVLLAIFTAGHLIGLYLYQFQFFQEAVPPNDYYA  305
AFC88283.1       226   VVTILLGSSGMMLPSLTSSVYFFVFLGLCTWWSWCRTFDPLLFSCLCVLLAIFTAGHLIGLYLYQFQFFQEAVPPNDYYA  305
XP_040520788.1   228   VVTILLGSAGMMVPSLTSAVYFFVFLGLCTWWSCCQAFDPLIFSCLCVLMAIFSAGHLIGLYLYQLQFFQEVVPPKDFYA  307
XP_062836599.1   224   VVTILLGATGMMLPSLTSAVYFFLFLGLCTWWSCCRSFDPLIFSCLCVLMAIFSAGHLIGLYLYQLQFFQEIIPPDDFYA  303
XP_021323952.1   257   VVTIMLGMTGVTLPSLSSAVYFFVFLGLCTWWSLCKTFDKLLFSCLCVLMAIFSAGHLIVLYINQFQFLQEAISPTDGYT  336

ADN28065.1       306   RLFGIKSVIQTDCASTWKIIVNPDLSWYHHANPILLLVMYYTLATLIRIWLQEPL---VQEEM---AKEDEGALDCSSNQ  379
AFC88283.1       306   RLFGIKSVIQTDCSSTWKIIVNPDLSWYHHANPILLLVMYYTLATLIRIWLQEPL---VQDEG---TKEEDKALACSPIQ  379
XP_040520788.1   308   RLFGVTSLTQTNCSSTWMIIKNQELHWYHHANPVLLLVMYYTLATLIRLWLQEPI--qVPEEEKSESREDDKEIPCSPIP  385
XP_062836599.1   304   RLFGVTSVIQTNCSSTWKIITNTKLYWYHHTSPMLILLLYFILATLIRLWLNEPAvqhVPDDDKSGNREEE--TDCHPNE  381
XP_021323952.1   337   SVFGISSIIYTDCSSTWKLAVRSGLKWHHFVNPIMILVLYYTLATLIRLWLQDPMm--MEDSEDGEVIEGNEAME-NNVH  413

ADN28065.1       380   NTAERRRSLWYATQYPTDERKLLSMTQDDYKPSDGLLVTVNGNPVDYHTIHPSLPIENGPAKTDLYTTPQYRWEPS-EES  458
AFC88283.1       380   ITAGRRRSLWYATHYPTDERKLLSMTQDDYKPSDGLLVTVNGNPVDYHTIHPSLPMENGPGKADLYSTPQYRWEPS-DES  458
XP_040520788.1   386   MTAERRRSLWYASHYTTDERKLLSMTQDEYKPSDVLLVTVNGNPADYHTIHPSLPLENGPAKADMYSTPQYKWEPS-DNM  464
XP_062836599.1   382   MTAERRRSLWYSSRYPTDERKIL-------------LVTVNGTPLDYQNMHPLRPIENGPANADIYSRPHYRWDPTvDDL  448
XP_021323952.1   414   HTSIRKRLLWWKAHQKTEERNLIS-TQDGYSTSEVLVVTSNGTSFDYVS---AVPIENGPVSLDIYSTPQYKVDQS-VEY  488

ADN28065.1       459   SEK    KEEEEDKREDSEGEGsQEEKRSVRMHAMVAVFQFIMKQSYICALIAMMAWSITYHSWLTFVLLIWSCTLWMIR  534
AFC88283.1       459   SEK    REEEEEEKEEFEEERsREEKRSIKVHAMVSVFQFIMKQSYICALIAMMAWSITYHSWLTFVLLIWSCTLWMIR  534
XP_040520788.1   465   SEK    EEEEVEEEEEVP----QEEKENVKLHALVSVFQFIMKQSYICALIAMMAWSITYHSWLTFVLLIWSCTLWMIR  536
XP_062836599.1   449   ETK    EEEEEQEEAEEEEDA--EEEKNSKVHAMVTVFHFIMKQSYVCALIAMMAWSITYHSWLTFVLLIWSCVLWMLR  522
XP_021323952.1   489   DEK[14]EEEEKTDEEEEAE----EEEQGGPPAALVKVFKFIMKQSYICALIAMMAWSITYVSWLTFVFLIWSCTLWMVR  574

ADN28065.1       535   NRRKYAMISSPFMVVYANLLLVLQYIWSFELP--EIKKVPGFLEKKEPG--ELASKILFTITFWLLLRQHLTEQKALREK  610
AFC88283.1       535   NRRKYAMISSPFMVVYGNLLLILQYIWSFELP--EIKKVPGFLEKKEPG--ELASKILFTITFWLLLRQHLTEQKALQEK  610
```

```
XP_040520788.1  537   DRRKYAMISSPFMVFYGNLLLILQYIWSIELKndELPQVSGFLERKEPG--ELASKILFTITFWLLLRQHLTEQKALQLK  614
XP_062836599.1  523   DRRKYAMISSPFMVVYGNLLLTLQYIWSIELDdhELPEVSDFLERKRPT--ELASKILFAITFWLLLRQHLTEQKALRLK  600
XP_021323952.1  575   DRRKYAMLTSPFMVAYGNFLLVLQYIWCFE----KLKPVSGFFVKMEVPftELSTKVLCQLSFWLLLRQTLMERQERKEE  650

ADN28065.1      611   EALLSEVKIGSQELEEKEDEELQ[4]EGEPTEKEEEEE   EE[13]EEVEEDDDQDIMKVLGNLVVALFIKYWIYVCGG  697
AFC88283.1      611   EALLSEVKIGSQENEEK-DEELQ[4]EGEP---KEEEE   EE[13]EEAEEEDEQDIMKVLGNLVVAMFIKYWIYVCGG  693
XP_040520788.1  615   EATLSEVKVGNEEDEEKEEGELQ   EGEVAEEEEEEE   EE     QEDDDEDEQDIMKVLGKLVMAMFIKYWIYVCGG  684
XP_062836599.1  601   EAVLSEVKVEEEGSEEKEGSQKE   EEESPKEEEEEE[6]EE     EEESEEEEEDVMKVLGNLVMAMFVKYWIYVCGG  676
XP_021323952.1  651   EAALSDIKV--------------   ------DEQKKV   EE     KSEEEVDENDLMQVLGKLVMALLVKYWIYVCGG  700

ADN28065.1      698   MFFFVSFEGKIVMYKIIYMVLFLFCVALYQVHYEWWRKILKYFWMSVVIYTMLVLIFIYTYQFENFPGLWQNMTGLKKEK  777
AFC88283.1      694   MFFFVSFEGKIVMYKIIYMVLFLFCVALYQVHYEWWRKILKYFWMSVVIYTMLVLIFIYTYQFENFPGLWQNMTGLKKEK  773
XP_040520788.1  685   MFFFVSFEGRIVMYKIIYMVLFLFCVALYQVHYEWWRKILKYFWMSVVVYTMLVLIFIYTYQFESFPGLWKNMTGLDENK  764
XP_062836599.1  677   MFFFVSFEGKIVMYKIIYMMLFLFCVVLYQVHYEWWRQILKYFWMSVVVYTMLVLVLIYTYQFDSFPTLWKNATGMNDEQ  756
XP_021323952.1  701   MFFFVSFEGDMATYKIIYMMMLLSCVALYQVHYEYWRRILKYFWMSVVVYTMLVLILIYTSQFEDGIPTWTRMTGLDKDV  780

ADN28065.1      778   LEDLGLKQFTVAELFTRIFIPTSFLLVCILHLHYFHDRFLELTDLKSIPSKEDNTIYSHAKVNGRVYLIIN    RLAHP  853
AFC88283.1      774   LEDLGLKQFTVAELFTRIFIPTSFLLVCILHLHYFHDRFLELTDLKSIPSKEDNTIY--------------    RLAHP  835
XP_040520788.1  765   LADLGLKRFSVAELFTRIFIPTSFLLACILHLHYFHDRFLQLTDLKAVTSKQDNTIYSHAKVNGRVYLIIN[11]ELVHQ  851
XP_062836599.1  757   LKDLGLEQFSVGELFTRIFIPTSFLLVCILHLHYFHDRFLQLTDLKAIISKQDSAIY--------------    RLVHQ  818
XP_021323952.1  781   LKDIGLEKFSLSNLFTRIFIPTSFLLVCILHLHYFHDHFLELTDLKAVVSKQESTIYSYAKVSGRIYLIVD[11]KLAHP  867

ADN28065.1      854   EGSLPDLAIMNMTASLDKPEVQKLAESGEERPEEC   ----VKKT---EKGEAGKDSDESEEEEDEEEESEEEE-SSDL  922
AFC88283.1      836   EGSLPDLTMMHLTASLEKPEVRKLAEPGEEKLEGY   ----SEKA---QKGDLGKDSEESEEDGEEEEESEEEEeTSDL  905
XP_040520788.1  852   DGSLPDITMMNLTASSEKEEDKMLKEAGEKRMEEP[1]GEGGKGRA---EKGKEEEEEDEEKEDDDVDDESEEEE-TTDL  925
XP_062836599.1  819   DGSLPDITMMTLHASIEKEEEKILQDQENMSQEQE[8]SEHEKEEN---ENEKEEEEGDEEEYD---EDESETEE-TSDL  896
XP_021323952.1  868   DGSLADLTIVSSSPEPLLKDEKEKESLNEVLVVDC[1]GEEKKKDSlsiYSAQMSSQEDVHQSSAATDPELPSEQ-SSEM  944

ADN28065.1      923   RNKWHLVIDRLTVLFLKFLEYFHKLQVFMWWILELHIIKIVSSYIIWVTVKEVSLFNYVFLISWAFALPYAKLRRAASSV  1002
AFC88283.1      906   RNKWHLVIDRLTVLFLKFLEYFHKLQVFMWWILELHIIKIVSSYIIWVSVKEVSLFNYVFLISWAFALPYAKLRRLASSV  985
XP_040520788.1  926   RNKWHLVIDRLTVLFLKFLEYFHKMQVFVWWLLELHIIKIVSSYIIWVTVKEVSLFNFVFLIAWALALPYAQFRPLASSI  1005
XP_062836599.1  897   RNKWHLVIDRLTVLFLKFLECFHKLQVFVWWILELHIIKIVSSYIIWVTVKEVSLLNYAFLIAWAFALPYSQFRPLASSV  976
XP_021323952.1  945   KNKWHLVVDRLTVLFLKFLEYFHKLKLFVWWLLEMHIIKIVATYIILLSVKEVSLLNFVFLISWAFALPYKQFRVLSSSV  1024

ADN28065.1      1003  CTVWTCVIIVCKMLYQLQTIKPENFSVNCSLPNENQTNIPLHELNKSLLYSAPVDPTEWVGLRK-SSPLLV-YLRNNLLM  1080
AFC88283.1      986   CTVWTCVIIVCKMLYQLQTIKPENFSVNCSLPNENQTNIPFNELNKSLLYSAPIDPTEWVGLRK-SSPLLV-YLRNNLLM  1063
XP_040520788.1  1006  CTVWTCVIIVCKMLYQLTSIDPSTFSSNCTLPRENETKVDLEELKTSVLYSGPVDPAEWVGLRK-SYPLLL-YLRNNLLM  1083
XP_062836599.1  977   CTVWTCVIIICKMFYQLESINTANFSVNCTMPN---TTVKPEVLSESLLYRKPIDPTEWVGLKK-SYPLLV-YLRNNLLM  1051
XP_021323952.1  1025  CTVWTCVIIVCKLFYQLKTINPANYSSDCIMPN--YTETEKKDMKTSLLYRDPVDPANWVGLRKfDHGIILpNLRNNLLM  1102

ADN28065.1      1081  LAILAFEVTVYRHQEYYRGRNNLTAPVSKTIFHDITRLHLDDGLINCAKYFVNYFFYKFGLETCFLMSVNVIGQRMDFYA  1160
AFC88283.1      1064  LAILAFEVTIYRHQEYYRGRNNLTAPVSRTIFHDITRLHLDDGLINCAKYFINYFFYKFGLETCFLMSVNVIGQRMDFYA  1143
XP_040520788.1  1084  LAILAFEVTIYRHQEYYRCRNNLTAPVTKTIFHDITRAHLDDGLVNCVKYFINYFFYKFGLETCFLLSVNVIGQRMDFYA  1163
XP_062836599.1  1052  LAILAFEVTIYRHQEYYRCRNNLTAPDTKTIFHDITRLHLDDGVISCLKYFINYFFYKFGLESCFLLSVNVIGQRMDFYA  1131
```

```
XP_021323952.1  1103  LALLAFEVTIYRHQEFFRLRNKLSPPPSRTIFHDITRQHLDNGIIECAKYFINYFFYKFGLEVCFLLAINVMGQRMDFYS  1182

ADN28065.1      1161  MIHACWLIGVLYRRRRKAIAEVWPKYCCFLACIITFQYFVCIGIPPAPCRDYPWRFKGAYFNDNIIKWLYFPDFIVRPNP  1240
AFC88283.1      1144  MIHACWLIAVLYRRRRKAIAEIWPKYCCFLACIITFQYFICIGIPPAPCRDYPWRFKGASFNDNIIKWLYFPDFIVRPNP  1223
XP_040520788.1  1164  MIHAFWLIAVLYRRRRKAIAEIWPKYCCFLACIITFQYFLCIGIPPAPCKDYPWRSGNANFNSNIIKWLYFPDFIVRPNP  1243
XP_062836599.1  1132  MIHAFALIVVLYRRRRKAIAEVWPKYCCFLACIITFQYFMCIGIPPAPCKDYPWRNPNSNFNSNIIKWLYFPDFIQRPNP  1211
XP_021323952.1  1183  MVHGLALAVVMFRRRRKAIAEIWPKYCCFLACMITFQYFICIGIPPAACADYPWRFPNSSMTSNVIKWLYLPDFHTPPSS  1262

ADN28065.1      1241  VFLVYDFMLLLCASLQRQIFEDENKAAVRIMAGDNVEICMNLDAASFSQHNPVPDFIHCRSYLDMSKVIIFSYLFWFVLT  1320
AFC88283.1      1224  VFLVYDFMLLLCASLQRQIFEDENKAAVRIMAGDNVEICMNLDAASFSQHNPVPDFIHCRSYLDMSKVIIFSYLFWFVLT  1303
XP_040520788.1  1244  VFLVYDFMLLLCASLQRQTFEDENKAAVRIMAGDNVEICMNLEAASFSQHNPVPDFIHCRSYLDMYKVIIFSYLFWFVLT  1323
XP_062836599.1  1212  VFLVYDFMLLLCASLQRQTFEDENKAAVRIMAGDNVEICMNLDAASFSQHNPVPDFIHCRSYLDMGKVIMFSYLFWFVLT  1291
XP_021323952.1  1263  MFLGYDFMLLLCASLQRQVFDEENKAAVRLMAGDNVEICRDLDAASFSVHNPVPDFIHCRSYLDMLKVIMFSYLFWFVLT  1342

ADN28065.1      1321  IIFITGTTRISIFCMGYLVACFYFLLFGGDLLLKPIKSILRYWDWLIAYNVFVITMKNILSIGACGYIGALVRNSCWLIQ  1400
AFC88283.1      1304  IIFITGTTRISIFCMGYLVACFYFLLFGGDLLLKPIKSILRYWDWLIAYNIFVITMKNILSIGACGYIGTLVHNSCWLIQ  1383
XP_040520788.1  1324  IIFITGTTRISIFCMGYLVACFYFLLFGGDLLLKPIRSILRYWDWLIAYNVFVITMKNILSIGACGYIESLIQNSCWLIQ  1403
XP_062836599.1  1292  IIFITGTTRISIFCMGYLIACFYFLLFGGDLLLKPIRSILRYWDWLIAYNVFVITMKNVLSIGACGYIGSLIKNSCWVIQ  1371
XP_021323952.1  1343  IIFITGTTRISIFCMGYLVACFYFLLFGGQLLLKPIKEILRYWDFLIAYNVFVITMKNVFAILACGYINSLVKNSCWLIQ  1422

ADN28065.1      1401  AFSLACTVKGYQMPED--DSRCKLPSGEAGIIWDSICFAFLLLQRRVFMSYYFLHVVADIKASQILASRGAELFQATIVK  1478
AFC88283.1      1384  AFSLACTVKGYQMPAA--NSPCTLPSGEAGIIWDSICFAFLLLQRRVFMSYYFLHVVADIKASQILASRGAELFQATIVK  1461
XP_040520788.1  1404  AFSLACTVKGYRIPTN--NADCKLPSGEAGIIWDSICFAFLLLQRRVFMSYYFLHVVADIKASQILASRGAELFQATIVK  1481
XP_062836599.1  1372  IFSLSCTVKGYEIPSN-kDSECKIPSGEAGIIWDSICFAFLLLQRRVFMSYYFLHVVADIKASQILASRGAELFQATIVK  1450
XP_021323952.1  1423  LLSLACTIKDYQPKTDlpGVDCELPKDEAGIIWDSICFTFLLLQRRVFMSYYFLHVIADIRAGQILASRGAELFQASIVK  1502

ADN28065.1      1479  AVKARIEEEKKSMDQLKRQMDRIKARQQKYKKGKERMLSLTQESGEGQDIQKVSEEDDEREADKQKAKGKKKQWWRPWVD  1558
AFC88283.1      1462  AVKARIEEEKKSMDQLKRQMDRIKARQQKYKKGKERMLSLTQEPGEGQDMQKLSEEDDEREADKQKAKGKKKQWWRPWVD  1541
XP_040520788.1  1482  AVKARIEEEKKSMDQLKRQMDRIKARQQKYKKGKERMLSMTQDSTEGPEIRKVSEEDDEGEADKEKAKGKKKLWWRPWVD  1561
XP_062836599.1  1451  AVKARIEEEKKSMDQLKRQMDRIKARQQKYKKGKERVLSMNQEASEGQAIQKEDDDDDDGEADKEKAKGKKKQWWRPWVD  1530
XP_021323952.1  1503  AVRARLEEEKKSMEQLKRQMDRIKTRQQKFKRGKEKMLSIAQESGDGEKLIPPVDNEEEEDEEKKKNKVQKKQWWRPWVD  1582

ADN28065.1      1559  HASMVRSGDYYLFETDSEEEEEEElKKEDEEPPRKSAFQ    FVYQAWITDPKTALRQRRKEKKKLAREEqKERRKGSG  1634
AFC88283.1      1542  HASMVRSGDYYLFETDSEEEEEEElKKEDEEPPRRSAFQ    FVYQAWITDPKTALRQRHKEKKRSAREErKRRRKGSK  1617
XP_040520788.1  1562  HASMVRSGNYYLFETDSEEEEEEE-KKEEEEPRKKSAFQ[33]FVYQAWITDPKTALRQRRKEKKSFQKEE-KRRRKVYG  1668
XP_062836599.1  1531  HASMVRSGDYYLFETDSEEEEEDE-KKEEEEPPKRSAFQ[33]FVYQAWITDPKTALRQRRKEKKKIEKEG-RRRRKGSG  1637
XP_021323952.1  1583  HASMVRSGDYYLFETDSEEEEEEE-EKKDEEPPKRSAFQ    FVYHTWIAESKAALKERGKGKRFWKRYG-RRLKKEKK  1656

ADN28065.1      1635  DGP--VE-WEDREDEPVKKKSDGPDNIIKRIFNILKFTWVLFLATVDSFTTWLNSISREHIDISTVLRIERCMLTREIKK  1711
AFC88283.1      1618  EGP--VE-WEDREDEPIKKKSDGPDNIIKRIFNILKFTWVLFLATVDSFTTWLNSISREHIDISTVLRIERCMLTREIKK  1694
XP_040520788.1  1669  DGSTDAD-CEDSEEEPVKKKSDGPDNIIKRIFNILKFTWVLFLATLDSFTAWLNSISREHIDISTVLRIERCMLTREIKK  1747
XP_062836599.1  1638  DVSIDVE-GED-REEPAKKKSDGPDNIIKRIYNILKFTWVLFLATIDSFTAWLNSISREHIDISTVLRIERCMLTREIKK  1715
XP_021323952.1  1657  EEHVAIEiGEDERQSSEEEKTDGPDNIIKRVFNIIKFTWALFLTTVESITKWLNSVCREYIDISTVLRIERCMLTREVKK  1736
```

```
ADN28065.1       1712  GNVPTRESIHMYYQNHI-MNLSRESGLDTIDEHSGAGSR   AQAAHRMDSLDS-     --RDS   ISSCYTEAT-LLI  1776
AFC88283.1       1695  GNVPTRESIHMYYQNHI-MNLSRESGLDTIDEHPGAASG   AQTAHRMDSLDS-     --HDS   ISSEPTQCT-MLY  1759
XP_040520788.1   1748  GNVPTRESIHMYYQNHM-MKLSKESGLDSIDKNPGQASG   LQTSERMDSLDSA     ASRDS   ISSCYTEAT-MLF  1815
XP_062836599.1   1716  GNVPTRESIHMYYQNHM-MKLSRESGLDEIYE-PGQA--   ----ERTTSLESA     ASRDS   ETSCCTEATtLLL  1777
XP_021323952.1   1737  GNVPSRESIHVYYQKQMkLNGSRESGLDRISEEDSASNR[9]LDSFASRDSISSA[45]SSADS[4]ISSEATQCV-TLF  1863

ADN28065.1       1777  SRQSTLDDLDGQDPVPKTSERARP   RLRKMFSLDMSSSSADSGS   VASSEPTQCTMLYSRQGTTETIEEVEAE     1847
AFC88283.1       1760  SRQGTTETIE--------------   --------EVEAEQEEEAG   STAPEPREA-------------KEYEAT     1795
XP_040520788.1   1816  SRQSTLDDLDGPDTVPKTSERARP   RLRKMQSMDMSSSSADSGS   IVSSEPTQVTMLYSRQGTTETIEEVEGE     1886
XP_062836599.1   1778  SRQSTLDDLDGPEPIPKTSERARP   RLRKMYSMDVSSSSADSGS   IVSSEATQITMLYSRQGTAETIEEVEGE     1848
XP_021323952.1   1864  SRQGTNDTIDEAEDEREQLEHKQK[4]ELRDKPEEEQEVEISDQGK[8]LVTQEVKEDVLPVSDQLTGEVTEEVEEE[5]  1951

ADN28065.1       1848  A-EEEVVE--GLEPElhDAEEKEyaaEYEAGVE     EISLTPDEELPQFST--DDCEAPPSYSKAVSFEHLSFA-SQDD  1917
AFC88283.1       1796  G-YDVGA----------------------MGAE     EASLTPEEELTQFSTLDGDVEAPPSYSKAVSFEHLSFG-SQDD  1847
XP_040520788.1   1887  H-EEEGAH--SASRQ--DEEEVE---DYSLGSE     GAAYTPDTDVPLYSTVDSNAEAPPSYSKAVSFEHLPFG-SPDD  1953
XP_062836599.1   1849  EpEEEGAD--PASRQ---EHDME---DYCLDSE     GAAYTPDTDMPSYSALDGIAEPPPSYSKVVSLELLPLDdSQDD  1916
XP_021323952.1   1952  EmEEEVEKeiGAKVEkeVEAEKEeeaEKEVGEE[39]GPLFTPDTDAPNTS----DADVPPSYSKAVSFDRLSVS-SDDS  2061

ADN28065.1       1918  SGAKNHMVVSPDDSRTDKLESSILPPLTHELTASDLLMSKMFHDDELEESEKFYVDQPRFLLLFYAMYNTLVARSEMVCY  1997
AFC88283.1       1848  SAGKNRMAVSPDDSRTDKLGSSILPPLTHELTASELLLKKMFHDDELEESEKFYVGQPRFLLLFYAMYNTLVARSEMVCY  1927
XP_040520788.1   1954  SAGKSLMMVSPDDSRTDRLNDAILPPLTHELTASELLLNKMFHDDELEESEKFYVGQPRVLLLIYALYNTLVARSEMVCY  2033
XP_062836599.1   1917  SSDKNQMMVSPDENQSIKLEDSILPPLTHELTASDLLVNKMFKDEELDESEKFYADQPRFLLLIYALYNTLVARSEMVCY  1996
XP_021323952.1   2062  DSDKRLMLMTPDSKSD--LDDPLLPSMTTDMTASELLLNKMFYDEMLDSSDRFYKSQPLGLQLCYALYNLLVAHSEMVCY  2139

ADN28065.1       1998  FVIILNHMTSASIITLLLPILIFLWAMLSVPRPSRRFWMMAIVYTEVAIVVKYFFQFGFFPWNKDLEIYKERPYFPPNII  2077
AFC88283.1       1928  FVIILNHMVSASMITLLLPILIFLWAMLSVPRPSRRFWMMAIVYTEVAIVVKYFFQFGFFPWNKNVEVNKDKPYHPPNII  2007
XP_040520788.1   2034  FVIILNHMISASMITLVLPILIFLWAMLSVPRPSKRFWMTAIVYTEVAIVIKYFFQFGFFPWNKYVDYTKDKPYHPPNII  2113
XP_062836599.1   1997  FVIILNHMISASMITLVLPILIFLWAMLSVPRPSKRFWMTAIVYTEVAIVIKYFFQFGFFPWNANVEIKKDKPDHPPNII  2076
XP_021323952.1   2140  LVIILNHMISASMATLVLPILIFLWAMLSVPRPSKRFWMTAIVYTEVTIVIKYFFQFSFFPFNQNLEVNKGKPYHPPNIL  2219

ADN28065.1       2078  GVEKKEGYVLYDLIQLLALFFHRSILKCHGLWDEDD   IVDSNTDKEGSDD-ELSL---DQGRRGSSD--SLKSINLAA  2148
AFC88283.1       2008  GVEKKEGYVLYDLIQLLALFFHRSILKCHGLWDEDD   MTESGMAREESDD-ELSL---GHGRRDSSD--SLKSINLAA  2078
XP_040520788.1   2114  GIEKKEGYVHYDLVQLLALFFHRSILKCHGLWDEDE   KGDNSSNKGDTDD-ELSL---PGGRRDSSG--SLKSVNLAA  2184
XP_062836599.1   2077  GIDKKEGYVHYDLVQLLALFFHRSILKCHGLWDDED   GGDNISSKDDSDD-ELA----ESDRRGSSD--SLTSVNLAT  2146
XP_021323952.1   2220  GTEKKDGYVHYDLVQLLALFFHRSILKCHGLWDEDD[4]RKDECPRQESVDDgKLSVvmsPDEDRKSSPaySLKSMNLGM  2300

ADN28065.1       2149  SVESVHVT   FPEQPAAIRRKRSCSSSQISPRSSFSSNRSKRGSTSTRNSSQKGSSVLS--LKQKSKRELYMEKLQEHL  2223
AFC88283.1       2079  SVESVHVT   FPEQQTAVRRKRSGSSSEPSQRSSFSSNRSQRGSTSTRNSSQKGSSVLS--IKQKGKRELYMEKLQEHL  2153
XP_040520788.1   2185  SVESIHVH   FPEQQTAIRRKSSSSASQLSHRSSFSSHRSKRGSTSTRNSSQKGSSVLS--IKQKSRKELLMEKFREQM  2259
XP_062836599.1   2147  SVESIHVH   FPEQQAAVRRKSSSSGSQLSHRSSHSSHRSKRGSTSTRNSSQKGSSVLS--IKQKSRKELLMEKLREQM  2221
XP_021323952.1   2301  SVDSSQVH[4]YPEQRPYLRRQST-SGSHFSHRS---SARSKRGSTSTRHSTHGENSTTEseVPQKSRKEMIMEKIREQL  2377

ADN28065.1       2224  IKAKAFTIKKTLQIYVPIRQFFYDLIHPDYSAVTDVYVLMFLADTVDFIIIVFGFWAFGKHSAAADITSSLSEDQVPGPF  2303
AFC88283.1       2154  IKAKAFTIKKTLEIYVPIKQFFYNLIHPEYSAVTDVYVLMFLADTVDFIIIVFGFWAFGKHSAAADITSSLSEDQVPGPF  2233
```

```
XP_040520788.1  2260  IKAKAFTIKKTLQVYVPIRQFFYNLIHPDYSAVTDVYVLMFLADTVDFIIIVFGFWAFGKHSAAADITSSLSEDQVPEAF  2339
XP_062836599.1  2222  ILAKAFTIKKTLQVYVPIKQFFYNLIHPDYSAVTDVYVLMFLADTVDFIIIVFGFGAFGKHSAAADITSSLSEDQVPQAF  2301
XP_021323952.1  2378  IKAKVYLLKRLVEFYQPIRQFFYNLVHPEYSAVTDVYVLMFLADTVDFIIIVFGFWAFGKHQGGADITSSLSEDQVPGPF  2457

ADN28065.1      2304  LVMVLIQFGTMVVDRALYLRKTVLGKVIFQVILVFGIHFWMFFILPGVTERKFSQNLVAQLWYFVKCVYFGLSAYQIRCG  2383
AFC88283.1      2234  LVMVLIQFGTMVVDRALYLRKTVLGKVIFQVILVFGIHFWMFFILPGVTERKFSQNLVAQLWYFVKCVYFGLSAYQIRCG  2313
XP_040520788.1  2340  LVMVLIQFGTMVVDRALYLKKTVMGKVIFQVILVFGIHFWMFFILPGVTERKFSQNTVAQLWYFVKCVYFGLSAYQIRCG  2419
XP_062836599.1  2302  LVMVLIQFGTMVVDRALYLRKTVMGKVIFQVILVFGIHFWMFFILPIVTERKFSKNTVAQLWYFVKCVYFGLSAYQIRCG  2381
XP_021323952.1  2458  LVMVLIQFGTMVVDRALYLRKTVVGKVIFQVILVFGIHFWMFFILPGITERRFSQNTIAQLWYFVKCIYFGLSAYQIRCG  2537

ADN28065.1      2384  YPTRVLGNFLTKSYNYVNLFLFQGFRLVPFLTELRAVMDWVWTDTTLSLSSWICVEDIYAHIFILKCWRESEKRYPQPRG  2463
AFC88283.1      2314  YPTRVLGNFLTKSYNYVNLFLFQGFRLVPFLTELRAVMDWVWTDTTLSLSSWICVEDIYAHIFILKCWRESEKRYPQPRG  2393
XP_040520788.1  2420  YPTRVLGNFLTKSYNYVNLFLFQGFRLVPFLTELRAVMDWVWTDTTLSLSSWICVEDIYAHIFILKCWRESEKRYPQPRG  2499
XP_062836599.1  2382  YPTRVLGNFLTKSYNYVNLFLFQGFRLVPFLTELRAVMDWVWTDTTLSLSSWICVEDIYAHIFILKCWRESEKRYPQPRG  2461
XP_021323952.1  2538  YPTRILGNFLTKSYNYANLFLFQGFRLIPFLTELRAVMDWVWTDTTLSLSSWICVEDIYAHIFILKCWRESEKRYPQPRG  2617

ADN28065.1      2464  QKKKKAVKYGMGGMIIVLLICIVWFPLLFMSLIKSVAGVINQPLDVSVTITLGGYQPIFTMSAQQSQLKVMDNSKYNEFL  2543
AFC88283.1      2394  QKKKKVVKYGMGGMIIVLLICIVWFPLLFMSLIKSVAGVINQPLDVSVTITLGGYQPIFTMSAQQSQLKIMDQQSFNKFI  2473
XP_040520788.1  2500  QKKKKVVKYGMGGMIIVLLICIVWFPLLFMSLIKSVAGITNKPLDVSITITLGGYQPIFTMSAQQSQLKDLNQTGFSAFL  2579
XP_062836599.1  2462  QKKKKAVKYGMGGMIIVLLICIVWFPLLFMSLIKSVAGVTNKPLDVSITITLGGYQPIFTMSAQQSQLRNMTGKEYSDFL  2541
XP_021323952.1  2618  QKKKKVVKYGMGGMIVMLLICIVWFPLLFMSLVKSVAGVVNKPLEVSVSLTLGGLQPIFTMSAQQNHIKSVTGAEFKKFT  2697

ADN28065.1      2544  KSFGPNSGAMQFLENYEREDVTVAELEGNSNSLWTISPPSKQKMIQELTDPNSCFSVVFSWSIQRNMTLGAKAEIATDKL  2623
AFC88283.1      2474  QAFSRDTGAMQFLENYEKEDITVAELEGNSNSLWTISPPSKQKMIHELLDPNSSFSVVFSWSIQRNLSLGAKSEIATDKL  2553
XP_040520788.1  2580  GSYRGNTAALQFLEGYGKEDITLADLEGNSNSLWTISPPSREKMIQGLLDFSAEFTVVLSWSIQRNLTLGAKAEIASDKL  2659
XP_062836599.1  2542  RKFKDNS-ALQFLENYGKEDITVAQLEGSSNSLWTISPPSRKNMIKELKANDSDFSLVISWSVQRNLSLGAKAEIASDKV  2620
XP_021323952.1  2698  DHYKSNANAIMFLESYFPEDLTIAHLEGSSNSLWTISPPSKENLKNMLNDTREPFPLTFTWSIQRNLSLGAKAETAMGKY  2777

ADN28065.1      2624  SFPLAVATRNSIAKMIA-GNDTES[4]VTIEKIYPYYVKAPSDSNSKPIKQLLSENNFMNITIILFRDN---VTKSNSEW  2700
AFC88283.1      2554  SFPLKNITRKNIAKMIA-GNSTES[4]VTIEKIYPYYVKAPSDSNSKPIKQLLSENNFMDITIILSRDN---TTKYNSEW  2630
XP_040520788.1  2660  TFGLPEKTRRDIATMMS-GKPLEK   VTLETVYPYYIKAPSDSLAKPIKQLLTDCRWENITVSLVKNV---SEEGVREW  2732
XP_062836599.1  2621  SLSLPINTRIKIAEMMEqSSSSNL   VTLEEVYPHYIKAPSDSVAKPIKQLLRKSKDENITVSLSKNTvsgNESDLREW  2697
XP_021323952.1  2778  FIELDDETRKGLTDQLG--NSSQR   VTIKKIFPRYIRAPSDSNAKPIDQLYNDGEYMDITLFLNKKN---SSEGVQEW  2849

ADN28065.1      2701  WVLNLTGSRIF-NQGSQALELVVFNDKVSPPSLGFLAGYGIMGLYASVVLVIGKFVREFFSGISHSIMFEELPNVDRILK  2779
AFC88283.1      2631  WVLNLTGNRIY-NPNSQALELVVFNDKVSPPSLGFLAGYGIMGLYASVVLVIGKFVREFFSGISHSIMFEELPNVDRILK  2709
XP_040520788.1  2733  WVLNQLGKR-Y-KTNEESLELFIFSDKVSPPSLGFLAGYGIMGLYASVVLVIGKFVREFFSGISHSIMFEELPNVDRILK  2810
XP_062836599.1  2698  WVLNQTYKH-L-DKDRTRLELIVFSDKVSPPSLGFLAGYGIMGLYASVVLVIGKFVREFFSGISHSIMFEELPNVDRILK  2775
XP_021323952.1  2850  WIVNQTDSGPLpGLNGSGLQIYIISDQVSPPSLGFLAGYGIMGLYMSVVLVIGKFVREFFSGISHTIMFEELPNVDRILK  2929

ADN28065.1      2780  LCTDIFLVRETGELELEEDLYAKLIFLYRSPETMIKWTREKTN  2822
AFC88283.1      2710  LCTDIFLVRETGELELEEDLYAKLIFLYRSPETMIKWTREKTN  2752
XP_040520788.1  2811  LCTDIFLVRETGELELEEDLYAKLIFLYRSPETMIKWTREKTN  2853
XP_062836599.1  2776  LCTDIFLVRETGELELEEDLYAKLIFLYRSPETMIKWTREKTN  2818
```

XP_021323952.1 2930 LCTDIFLVRETGELDLEEDLYAKLIFLYRSPETMIKWTREKSE 2972

Figure S3: Multiple Alignment of Canine (XP_038528610.1), humans (AFC88283.1), mice (ADN28065.1), green anole (XP_062836599.1), chicken (XP_040520788.1) and zebrafish (XP_021323952.1) Piezo2 result.

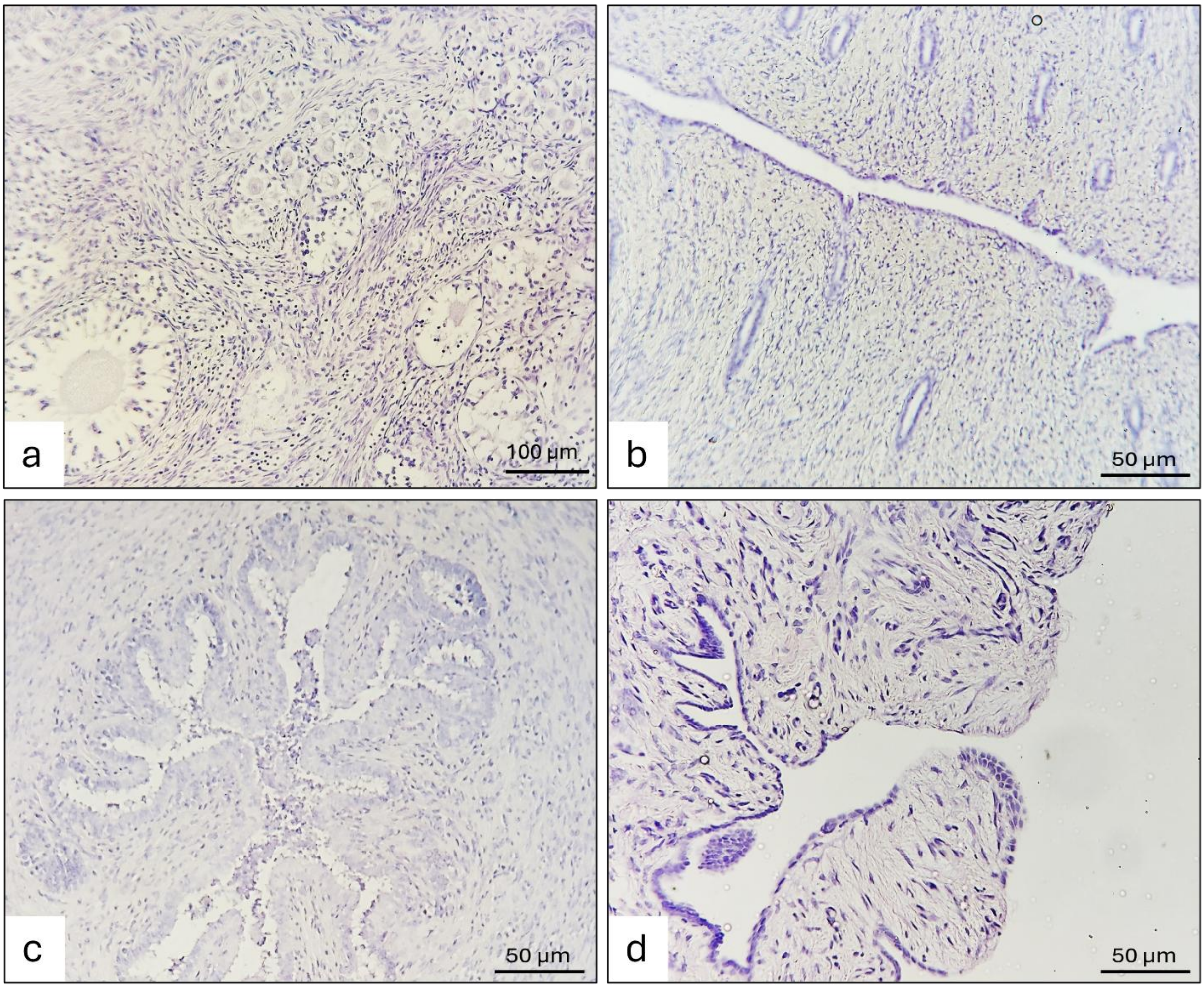

Figure S4. Representative images of the control reaction conducted omitting the primary antibodies ASIC2, ASIC4, PIEZO2. No immunoreactivity was observed. (a) ovary; (b) uterus; (c) isthmus; (d) fimbriae. Magnification40X
